# Combinatorial targeting of a specific EMT/MET network by macroH2A variants safeguards mesenchymal identity

**DOI:** 10.1371/journal.pone.0288005

**Published:** 2023-07-11

**Authors:** Dimitrios Valakos, Eleftheria Klagkou, Antonis Kokkalis, Alexandros Polyzos, Fotis L. Kyrilis, Aggelos Banos, Giannis Vatsellas, Maria Pliatska, Ethan Ford, Dimitrios J. Stravopodis, Dimitris Thanos

**Affiliations:** 1 Biomedical Research Foundation, Academy of Athens, Athens, Greece; 2 Section of Biochemistry and Molecular Biology, Department of Biology, School of Science, National and Kapodistrian University of Athens (NKUA), Zografou, Athens, Greece; 3 Section of Cell Biology and Biophysics, Department of Biology, School of Science, National and Kapodistrian University of Athens (NKUA), Zografou, Athens, Greece; University of Vermont, UNITED STATES

## Abstract

Generation of induced pluripotent stem cells from specialized cell types provides an excellent model to study how cells maintain their stability, and how they can change identity, especially in the context of disease. Previous studies have shown that chromatin safeguards cell identity by acting as a barrier to reprogramming. We investigated mechanisms by which the histone macroH2A variants inhibit reprogramming and discovered that they work as gate keepers of the mesenchymal cell state by blocking epithelial transition, a step required for reprogramming of mouse fibroblasts. More specifically, we found that individual macroH2A variants regulate the expression of defined sets of genes, whose overall function is to stabilize the mesenchymal gene expression program, thus resisting reprogramming. We identified a novel gene network (MSCN, mesenchymal network) composed of 63 macroH2A-regulated genes related to extracellular matrix, cell membrane, signaling and the transcriptional regulators Id2 and Snai2, all of which function as guardians of the mesenchymal phenotype. ChIP-seq and KD experiments revealed a macroH2A variant-specific combinatorial targeting of the genes reconstructing the MSCN, thus generating robustness in gene expression programs to resist cellular reprogramming.

## Introduction

Cellular reprogramming of Mouse Embryonic Fibroblasts (MEFs) towards pluripotency by OSKM (Oct4, Sox2, Klf4 and c-Myc) overexpression requires Mesenchymal to Epithelial Transition (MET) occurring during the first 3–4 days of reprogramming [1–3]. MET integrates a wide range of morphological and functional changes, all of which are orchestrated by transcriptional events, triggered by the OSKM [4]. These include alterations in the DNA methylation profile [5], recruitment of chromatin modifying enzymes [6] and repression/activation of transcriptional regulators [7–9].

Expression of OSKM in cells with mesenchymal characteristics (e.g. fibroblastic and hematopoietic) results in down-regulation of the master mesenchymal regulators Snai1/2, Twist1/2, Zeb1/2 and the mesenchymal surface proteins Thy-1, CD44 and N-Cad (Cdh2), while they up-regulate epithelial markers like E-Cad (Cdh1), Epcam and SSEA-1 (Fut4) and epithelium-related transcription factors like Irf6 and Ovol1 [8, 10, 11]. Various mesenchymal features including the elongated cell shape and interactions with the extracellular cell matrix are abandoned leading to a more rounded cell morphology, the acquisition of tight cell-cell junctions and apico-basal polarity [3]. These morphological alterations are accompanied by reorganization of the cytoskeleton, rebuffering of cell metabolism towards a more glycolytic program [11] and expression of various microRNAs like miR-200 and miR-181 [5, 12, 13], which promote MET in E-Cad-negative cells.

Genome-wide chromatin remodeling is a hallmark of reprogramming in order to support the dramatic changes of the transcriptome required for the generation of iPSCs. Histone variants contribute to chromatin and transcriptional complexity by assembling highly specialized nucleosomes with unique biochemical characteristics [14, 15]. The macroH2A (mH2A) family of histone variants consists of an H2A-like region, followed by a linker concluding in a globular macro domain [16]. This variant is encoded by two distinct genes in mammals. H2AFY encodes for the macroH2A1 variant, which is found in two different isoforms, mH2A1.1 and mH2A1.2 produced by alternative splicing [17], whereas the H2AFY2 gene encodes for the mH2A2 histone variant [18].

mH2A nucleosomes have diverse and pleiotropic molecular functions implicated in numerous cellular processes such as organization of chromatin structure, DNA damage repair, NAD+ metabolism, cancer progression, and cellular reprogramming [19]. Although mH2A proteins were initially linked to X-chromosome inactivation and general repressive effects on transcription [20], recent studies have demonstrated a positive role of mH2A in activation of transcription [21–24]. The unusual mH2A structure has suggested that its bivalent functions in gene expression could be due to alterations of chromatin structure or to promoter context and/or to interactions with other transcriptional regulatory proteins [23, 25]. In cases where mH2A nucleosomes mask activator binding sites, the underlying genes are repressed, whereas masking of repressor binding sites correlates with de-repression [23]. These effects are associated with increased stability of the heterotypic mH2A-H2B-containing nucleosomes as compared to canonical H2A-H2B-containing nucleosomes [26], thus resulting in a significant reduction of transcriptional noise [23].

By reducing transcriptional noise, mH2A nucleosomes stabilize gene expression programs and therefore are expected to resist to changes of cell identity. Thus, it may not be surprising that they act as barriers of cellular reprogramming. Knockdown (KD) of mH2A increases reprogramming efficiency both in mouse and human cells [27–30]. Furthermore, mH2A1 was shown to block cellular reprogramming by trapping reprogrammable cells at MET [30]. Accordingly, another study showed that mH2A also blocks EMT (Epithelial to Mesenchymal Transition), the reverse process of MET, upon initiation of cancer cell metastasis [31].

Herein, we performed KDs for individual mH2A variants in MEFs and Embryonic Stem Cells (ESCs), corresponding to the starting and ending stage analogs of cellular reprogramming, followed by deep transcriptome and computational analysis to examine their effects on MET. We integrated chromatin immunoprecipitation sequencing (ChIP-seq) with deep transcriptome sequencing (RNA-seq) experiments together with extensive Pathway and Network Analyses, and have unveiled a putative gene network operating in mesenchymal cells (Mesenchymal Network, MSCN), whose overall function is to safeguard the mesenchymal phenotype, thus resisting cellular reprogramming. MSCN is assembled from critical cell structure components, enzymes, transcriptional regulators and effector molecules, whose integrated expression is controlled by mH2A-containing nucleosomes, which are linked to the establishment and preservation of the mesenchymal phenotype.

## Results

### mH2A variants regulate the expression of MET/EMT genes in both MEFs and ESCs

Τo systematically investigate the role of mH2A variant-containing nucleosomes in the expression pattern of cellular reprogramming-related genes, we examined the RNA expression profile of MEFs (starting point of reprogramming) and ESCs (equivalent to iPSCs-end point of reprogramming) in the presence or absence of mH2A variant-specific shRNAs via RNA-seq analysis (S1A Fig). We carried out lentiviral-based shRNA KD assays (S1B Fig) in at least three biological replicates (since all mH2A variants are expressed in both MEFs and ESCs–S1C Fig) and verified that the reprogramming efficiencies of the KD cells, as compared to scramble shRNA cells, were increased significantly (S1D Fig). We hypothesized that this mH2A-driven effect is due to a decreased abundance and /or destabilization of mH2A-containing nucleosomes in the KD cells, and this affects the expression pattern of the underlying genes involved in the execution of cellular reprogramming pathways.

**Fig 1:**
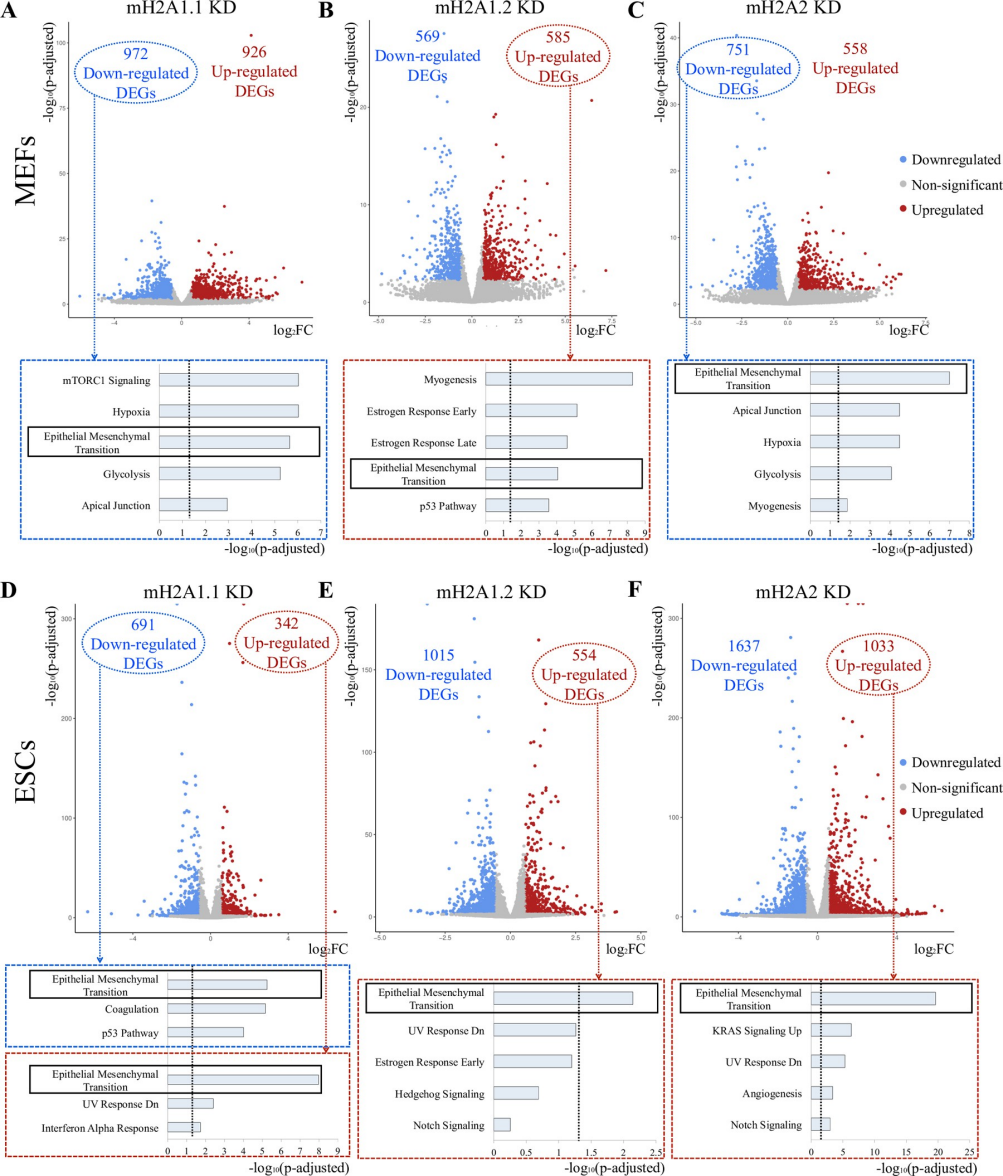
Effects of individual mH2A-variants KD in MEFs and ESCs. A. Volcano plot depicting the number of differentially expressed genes (DEGs) in mH2A1.1 KD MEFs, as compared to scramble cells. The Bar graph shown at the bottom of the plot indicates the Pathway Enrichment Analysis (Molecular Signature Database) for the indicated group of the up- or down-regulated genes. Cut-offs for Volcano plot: p-adjusted<0.05 and Log_2_FC > 0.58 or <-0.58. Cut-off for Pathway analysis: p-adjusted<0.05. **B.** Same as in (A), but for mH2A1.2 KD in MEFs. **C.** Same as in (A), but for mH2A2 KD in MEFs. **D**. Same as in (A), but for mH2A1.1 KD in ESCs. **E.** Same as in (A), but for mH2A1.2 KD in ESCs. **F.** Same as in (A), but for mH2A2 KD in ESCs.

To test this possibility, we performed RNA-seq analysis in mH2A KD MEFs and ESCs. Fig 1 depicts the transcriptome-wide alterations induced in MEFs and ESCs following individual mH2A variant KD, as compared to control cells transduced with a lentivirus expressing scramble shRNA. Overall, mH2A1.1 KD caused the most robust effects on the transcriptome in MEFs (1,898 differentially expressed genes-DEGs), as compared to mH2A1.2 (1,154 DEGs) and mH2A2 KDs (1,309 DEGs) (Fig 1A–1C), whereas mH2A2 KD was the most effective in altering the transcriptome in ESCs (2,670 DEGs) (Fig 1D–1F). We also noted that there is an approximately similar number of up- and down- regulated genes by any of the mH2A variants KD in MEFs. However, this is not the case in ESCs, where most of the genes appear to be positively regulated by mH2As (Fig 1). These observations suggest that individual mH2A variants differentially control the expression of target genes in MEFs and ESCs, thus suggesting that they possess tissue-specific gene regulatory functions.

Next, we carried out pairwise comparisons to identify DEGs that are commonly or specifically regulated by individual mH2A variants. We found that in MEFs, the majority of both the up- and down-regulated DEGs are affected by singular mH2A variants, whereas a smaller number of genes is affected by any of the pairwise combinations or from all three mH2As (S2A, S2B and S2E Fig). Collectively, these data demonstrate a division of labor among individual mH2A variants to control gene expression in MEFs. By contrast, in ESCs, we found that mH2A1.1 and mH2A1.2, but not mH2A2, share a significantly larger number of common target genes (S2C–S2E Fig).

On the surface, our finding that mH2A variants target mostly distinct sets of genes in MEFs is inconsistent with the fact all three variants can inhibit reprogramming with comparable efficiencies (S1D Fig). Notably, we have previously shown that mH2As block reprogramming by inhibiting the MET process [30]. Therefore, we searched our DEG lists for the presence of genes previously known to be involved in the MET or EMT pathways. Indeed, our unbiased Pathway Analysis revealed that MET/EMT are among the top enriched biological processes affected by mH2A-regulated genes. For example, many genes that are down-regulated by mH2A1.1 KD (Fig 1A) or up-regulated by mH2A1.2 KD (Fig 1B) in MEFs, appear to control the MET/EMT pathway, thus implying an unprecedented distinct mechanistic role of these splicing variants in regulation of the same pathway but in a different manner. Furthermore, mH2A2 KD revealed that MET/EMT was also the top enriched biological pathway of the down-regulated genes (Fig 1C).

Similar to MEFs, the MET/EMT pathways were significantly enriched in the up-regulated genes following mH2A1.1, mH2A1.2 and mH2A2 KDs in ESCs (Fig 1D–1F), a result further supporting their role in repressing differentiation-specific genes, thus maintaining the pluripotency phenotype. For example, in the case of mH2A1.1 KD, both up- and down-regulated genes are enriched for the MET/EMT pathway, and this effect is more robust for the up-regulated genes (Fig 1D). In summary, our data demonstrate that all mH2A variants regulate the MET/EMT pathway by inducing various molecular effects on the transcriptome.

**Fig 2:**
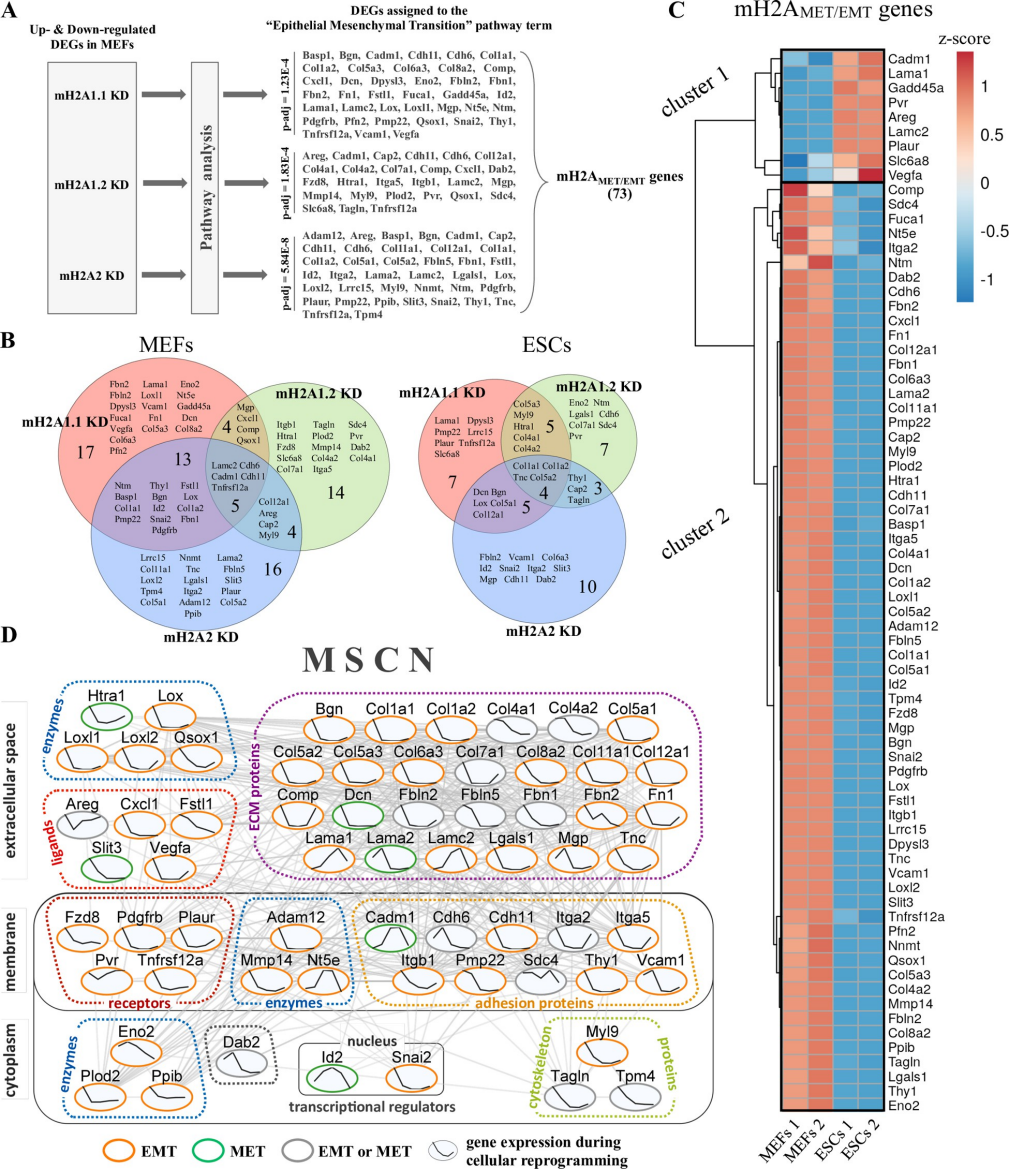
Identification of 73 mH2A_MET/EMT_ genes. **A.** Diagrammatic representation of the rational followed for the identification of the 73 mH2A_MET/EMT_ genes. **B.** Venn Diagram depicting the number and the identity of the commonly and differentially affected expression of the mH2A_MET/EMT_ genes following individual mH2A KDs in MEFs (left panel) and ESCs (right panel). Each Venn diagram was constructed using the differentially expressed genes (DEGs) defined with p-adjusted<0.05 and log_2_FC>0.58, or log_2_FC<-0.58. **C.** Heatmap depicting the expression levels (z-score) of the 73 mH2A_MET/EMT_ genes in MEFs and ESCs (n = 2). **D.** Reconstruction of a mH2A-regulated gene network safeguarding the mesenchymal cell identity. The mH2A-regulated gene network in MEFs (MSCN) was reconstructed from 63 out of the 73 mH2A_MET/EMT_ genes. The nodes were placed and grouped according to their known predominant subcellular localization (GO cellular component data) and molecular function (as annotated on the figure). Each connection (line) represents putative interactions and/or links between the indicated nodes. The expression trajectory of individual genes (nodes) during cellular reprogramming is depicted as a line within each node (Day 0, Day 3, Day 6, Day 9 and ESCs), according to our previous data [8] and publicly available data [32]. The border color of each node depicts the role assigned to this gene product in MET or EMT (orange: genes related exclusively, or mostly to EMT/mesenchymal phenotype, green: genes related exclusively, or mostly to MET/epithelial phenotype and grey: genes related to both EMT/mesenchymal and MET/epithelial phenotypes.

Since MET/EMT appears to be the prominent biological pathway affected by each of the mH2As KD, we mined the mH2A-dependent DEGs in MEFs and identified 73 genes that were previously known to be essential for the execution of the MET/EMT process (S1 Table). We annotated these genes as mH2A_MET/EMT_ genes (Fig 2A and S1 Table). Next, we carried out pairwise comparison analyses of the mH2A_MET/EMT_ genes and found that these genes are typically regulated by distinct mH2A variants, a result that is in agreement with the analysis shown in S2A and S2B Fig. More specifically, Fig 2B shows that, in MEFs, out of the 73 mH2A_MET/EMT_ genes, only 5 genes are regulated by all three mH2A variants, whereas, surprisingly, we found that only 4 genes are controlled by the mH2A1 splicing variants mH2A1.1 and mH2A1.2. On the contrary, mH2A1.1 and mH2A2 affect a larger number of common genes (Fig 2B, left panel). In ESCs, only 4 genes are regulated by all three mH2A variants, and contrary to the MEFs, only 5 genes are commonly regulated by both mH2A1.1 and mH2A2. In summary, these data demonstrate that the three mH2A variants regulate the MET/EMT pathway in a combinatorial fashion by targeting largely distinct sets of genes.

Next, we hypothesized that if these mH2A-regulated DEGs are indeed functionally related to MET/EMT and to cellular reprogramming, then they should display different, if not opposite, expression patterns between MEFs and ESCs. In other words, our prediction is that these genes should play a role in defining the mesenchymal and epithelial phenotype of MEFs and ESCs, respectively, and should change their expression pattern during reprogramming (MET) in order to facilitate the transition to pluripotency. Indeed, as shown in Fig 2C, the mH2A_MET/EMT_ genes form two distinct expression clusters. Cluster 1 is composed of a small number of genes which are up-regulated in ESCs and down-regulated in MEFs. By contrast, the larger Cluster 2 is built from genes which are upregulated in MEFs and down-regulated in ESCs (Fig 2C). As predicted from our hypothesis, Cluster 1 genes are mostly involved in promoting the epithelial phenotype, that is, the transition to and/or maintenance of pluripotency, whereas Cluster 2 genes are involved in establishing and/or maintaining the mesenchymal phenotype of MEFs (S1 Table). The observed opposite expression patterns of Clusters 1 and 2 between MEFs and ESCs further validates our results demonstrating the reverse effects caused by mH2A1.1 and mH2A2 KDs in the expression of genes of the MET/EMT pathway (compare Fig 1A and 1D with Fig 1C and 1F). These findings are also independently confirmed by the unbiased unique and separate clustering of these genes based on their expression patterns in MEFs (S2F Fig) and ESCs (S2G Fig). Of note, in ESCs, mH2A2 depletion leads to an extensive de-repression as compared to MEFs (S2G Fig).

### Reconstruction of a mH2A-regulated gene network that safeguards the mesenchymal cell identity

Although our studies on the role of mH2A nucleosomes in cellular reprogramming provide various functional and biological relevant insights, they do not present a global systemic view of how mH2A nucleosomes can coordinate the expression of both positively and negatively regulated genes, whose integrated function is to resist reprogramming, thus safeguarding the mesenchymal phenotype of MEFs. To uncover the functional hierarchy of the 73 mH2A_MET/EMT_ genes and to provide a comprehensive view of their biological roles in reprogramming, we integrated our RNA-seq experiments with previous cell reprogramming transcriptomics analyses [8, 32] to feed publicly available gene/protein-network databases. Interestingly, we found that 63 out of the 73 mH2A_MET/EMT_ genes can reconstruct a mH2A-dependent single gene network (mesenchyme network, MSCN), signifying various functional interconnections between the nodes (mH2A-regulated genes) and their topological linkage in the form of a dense, interconnected molecular circuit of interactions (Fig 2D). The expression pattern of individual gene network components (nodes) during the 18 day-long reprogramming process is diagrammatically depicted as a line graph within each node. Furthermore, the subcellular localization of each of the proteins encoded by the corresponding genes of the network is also depicted in Fig 2D. Overall, the mH2A_MET/EMT_ gene products are known to control several cellular properties such as attachment to various substrates, cell polarity, cell interactions and shape (S1 Table).

Focusing on key connections, we show that the network is reconstructed from protein nodes localized within all major cellular compartments, thus interconnecting the regulatory transcriptional activities of regulatory factors (e.g., Id2 and Snai2) with cytoplasmic enzymes and cytoskeleton proteins, cell membrane receptors, enzymes, adhesion molecules and various components of the extracellular space (Fig 2D). Notably, as shown in Fig 2D, the majority of the mH2A_MET/EMT_ gene products assemble a robust and remarkably dense extracellular matrix network including structural proteins involved in cell attachment (e.g. Cdh6, Cdh11, Thy1 etc.), enzymes that can modify extracellular components (Loxl1, Loxl2 etc.) and various ligands that can interact with the cell membrane (Vegfa, Cxcl1 etc.). The overall MSCN architecture perfectly highlights the prerequisite for extensive extracellular microenvironment and morphological modifications that must occur to initiate the reprogramming process. These observations further support our finding that MSCN plays a role in the conversion of mesenchymal to epithelial cells (S1 Table), a process that requires extensive cell-morphology alterations and detachment from the cellular substrate. Furthermore, as shown in Fig 2D, mH2A nucleosomes affect multiple signaling pathways, like PDGF, VEGF, WNT and EGF by adjusting the expression of their ligands including Vegfa, Cxcl1 and Areg, and receptors like Pdgfrb and Fzd8, whereas mH2A-dependent fine tuning in the expression of genes like Cdh6, Cadm1, and Cdh11, can affect intercellular interactions. In summary, the overall architecture of MSCN sheds light towards a better understanding of the complexity of the various intra- and inter-cellular molecular interactions occurring during the first days of reprogramming.

Our computational and experimental analyses revealed that nearly all genes building the MSCN are constitutively expressed in MEFs, and their expression is significantly reduced during reprogramming (Fig 2D), thus validating our view that MSCN safeguards the mesenchymal phenotype. Indeed, these genes encode proteins involved in the establishment and maintenance of the mesenchymal phenotype, and include among others 12 collagen genes (e.g. Col1a1, Col1a2, Col4a1, Col4a2, Col5a1, Col5a2, Col5a3, Col6a3, Col7a1, Col8a2, Col11a1 and Col12a1), the mesenchymal marker Thy1, Lysyl oxidases such as Lox, Loxl1 and Loxl2 that modify proteins of the extracellular matrix hence preserving the mesenchymal phenotype, and a plethora of other proteins like Vegfa, Plaur, Fbn2, Fn1, Cdh11, Mmp14, Adam12 and Pdgfrb that function to promote EMT, that is, to establish and maintain the MEF mesenchymal phenotype (S1 Table). Interestingly, we found that the gene expression patterns of the mH2A-regulated transcriptional regulators Id2 and Snai2 follow opposite expression routes during reprogramming (Fig 2D). More specifically, Id2 expression is transiently turned on, whereas Snai2 expression is progressively decreased during reprogramming (Fig 2D). These expression patterns are fully consistent with their biological functions, since Id2 suppresses EMT [33, 34], whereas Snai2 promotes EMT by activating the expression of mesenchyme-specific genes [35, 36]. Collectively, these data suggest that MSCN safeguards the mesenchymal identity in MEFs and is mainly regulated by mH2A variants, either in a direct or indirect manner.

### mH2A1.1 is the main variant regulating mH2A_MET/EMT_ gene expression and the MSCN

To examine whether the effect of mH2A KDs in the expression of the 73 mH2A_MET/EMT_ genes is a direct or an indirect effect mediated by other genes controlled by mH2As, we carried out ChIP-seq experiments using our mH2A variant-specific antibodies (see also S1E Fig) in MEFs and ESCs. The tornado plots shown in S3A Fig demonstrate specific binding of mH2A nucleosomes to the mouse genome and reveal that mH2A1.1 nucleosomes bind stronger to the genome as compared to mH2A1.2 and mH2A2, especially in ESCs (S3A, compare lower left panel with the rest of the panels).

We found that in MEFs, 46 of the 73 mH2A_MET/EMT_ genes are occupied by mH2A nucleosomes (S3B and S3C Fig, left panels) at regions spanning -10 kb to +10 kb from the TSS. Our analysis also revealed that mH2A1.1 and mH2A2 nucleosomes bind individually to a significantly higher number of mH2A_MET/EMT_ genes (17 and 14 genes respectively) as compared to 3 genes bound by mH2A1.2 alone (S3B Fig, left panel). Notably, the transcription regulators Id2 and Snai2, are direct targets of both mH2A1.1 and mH2A2 but not of mH2A1.2. In ESCs, 57 out of 73 mH2A_MET/EMT_ genes are occupied by mH2A-bearing nucleosomes (S3B and S3C Fig, right panels). Again, mH2A1.1 is the prevalent variant binding directly to the mH2A_MET/EMT_ genes. Interestingly, nearly all mH2A1.2 and mH2A2 targets are also bound by mH2A1.1 (S3B Fig, right panel).

Previous studies have provided evidence for a regulatory role of mH2A nucleosomes bound at proximal DNA regulatory regions or at the gene bodies of their targeted genes [22, 37]. Therefore, we performed Average Binding Analysis to test for the binding of mH2As at the regions located 5 kb upstream from TSS and at gene bodies of the mH2A_MET/EMT_ genes. mH2A1.1 is found to be the prevalent mH2A variant bound in both MEFs and ESCs followed by mH2A1.2 and mH2A2 (S3C Fig). These observations, in combination with the mH2A variant-specific KD effects (Fig 2B), raised the question whether mH2A nucleosome binding correlates directly to the expression of the underlying genes. We intersected the data shown in Fig 2B with those of S3B Fig and found that mH2A variants can indeed directly regulate the expression of many of the mH2A_MET/EMT_ genes (S3D Fig). In MEFs (S3D Fig, left panel), we found that most of the genes bound by mH2As changed their expression upon mH2A variant-specific KD, especially genes that are bound by combinations of different variants (e.g Lamc2, Cdh6, Tnfrsf12a). However, in ESCs (S3D Fig, right panel), this effect is less pronounced, presumably due to the significantly low number of mH2A_MET/EMT_ genes expressed in these cells. Taken together, these data suggest that the binding of mH2A-bearing nucleosomes to the mH2A_MET/EMT_ genes in MEFs results in a complex network of both direct and indirect functional consequences culminating in the inhibition of cellular reprogramming.

### mH2A1.1 and mH2A2 nucleosomes preserve the steady state of the MSCN network through direct binding at the transcriptional regulators’ Id2 and Snai2 genes

**Fig 3:**
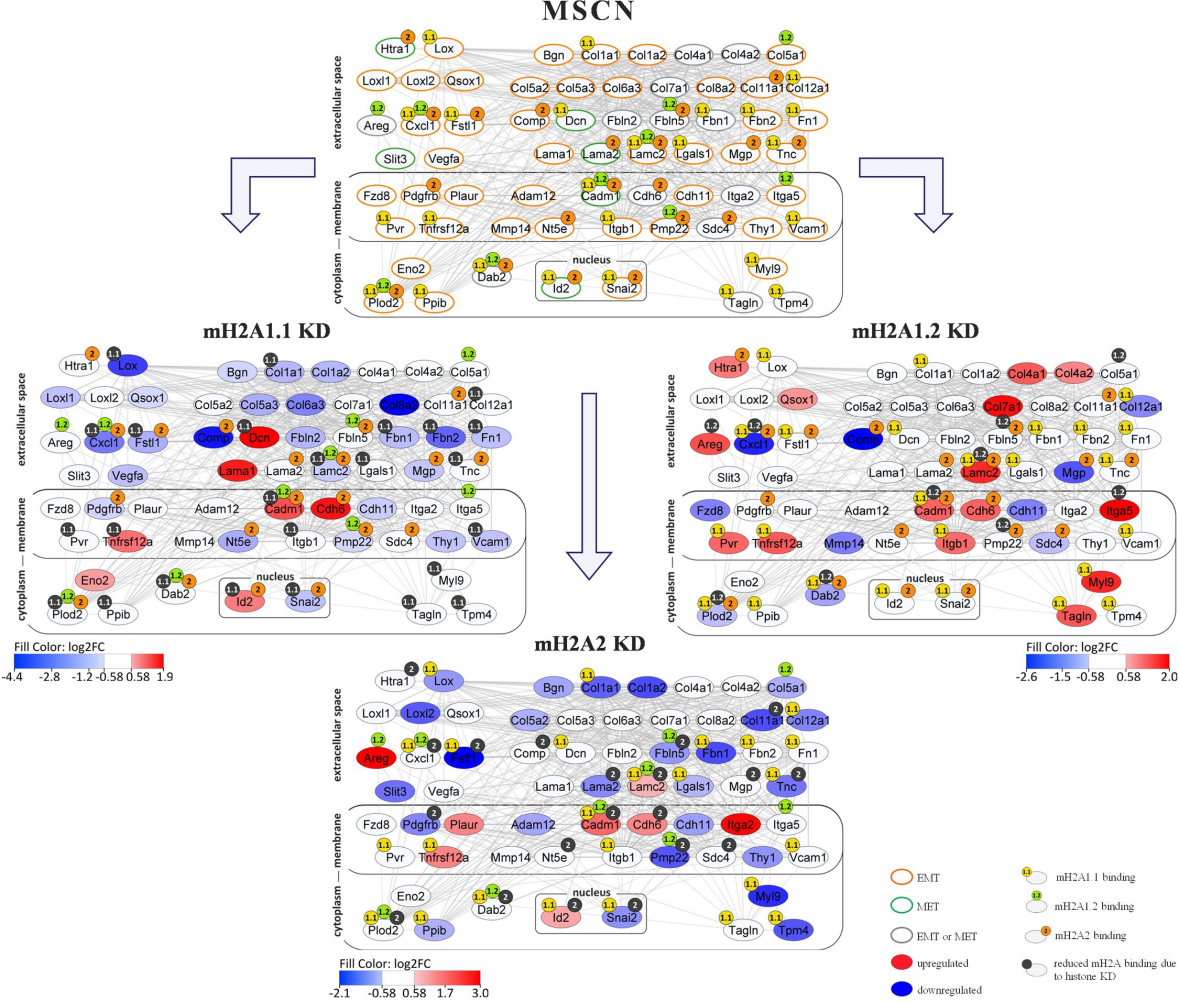
Diagrammatic representation of mH2A individual variants binding at the MSCN genes and the effect of the mH2A individual variants KD on the expression status of the network’s nodes. Top Panel: The mH2A-regulated MSCN network in MEFs. The node border color depicts the role of the corresponding gene regarding MET or EMT. The 1.1 or 1.2 or 2-related beads placed on the top of the nodes denote the direct mH2A1.1, mH2A1.2 or mH2A2 binding, respectively, as deciphered from our ChIP-seq binding profiles in MEFs. mH2A1.1-nucleosome binding appears in yellow color beads on the left side of the nodes, mH2A1.2-nucleosome binding is shown in green on the middle and mH2A2-nucleosome binding is depicted in orange on the right side of the nodes. Targets were defined using broad peaks as derived from SICER2 peak-calling analysis and the peaks were annotated to genes using GREAT tool. Left Panel: Shown is a schematic representation of the mH2A1.1 KD effects on the expression on each of the mH2A_MET/EMT_ genes as determined by RNA-seq analyses in MEFs. Blue-shade color denotes down-regulation (log_2_FC<-0.58), red-shade color denotes up-regulation (log_2_FC>0.58), whereas white color indicates no statistically significant changes in gene expression (p-adjusted<0.05). The mH2A1.1-nucleosome binding appears in dark grey to represent the reduced binding due to the histone KD. Right Panel: Same as in left panel, except that it represents the effect of mH2A1.2 KD. The mH2A1.2-nucleosome binding appears in dark grey to represent the reduced binding due to the histone KD. Bottom Panel: Same as in left panel, except that it represents the effect of mH2A2 KD. The mH2A2-nucleosome binding appears in dark grey to represent the reduced binding due to the histone KD.

In order to obtain a better picture of the functional role of mH2A variants in the expression of the MSCN genes and subsequently in the robustness of the network, we expanded the network shown in Fig 2D, by integrating the RNA-seq data from KD experiments with the ChIP-seq data presented above (Fig 3).

As seen in Fig 3, KD of individual mH2A variants can profoundly alter network integrity and equilibrium by upregulating or downregulating its building blocks (compare top panel with left, right and bottom panels). Evaluation of the genes affected by individual mH2A variants reveals common and mH2A variant-specific effects. In general, mH2A1.1 and mH2A2 show the most dramatic effects regarding the putative integrity of the network, since their KDs result in the most pronounced changes in the expression of the indicated MSCN genes. As predicted by the analysis shown in Fig 1, mH2A1.1 and mH2A2 KD cause down-regulation of the most MSCN genes. By contrast, mH2A1.2 KD results in the up-regulation of its targets (compare left and bottom panels with the right panel in Fig 3). Apart from the extensive down-regulation of their target genes, the mH2A1.1 and mH2A2 KD led to Id2 up-regulation and Snai2 down-regulation. Both genes are normally occupied by mH2A1.1 and mH2A2 nucleosomes (Figs 3 and S4A). This is an important finding because Id2 inhibits Snai1 and both are critical MET/EMT regulators [33–35, 38]. These data indicate that mH2A1.1 and mH2A2 regulate directly the expression of Id2 and Snai2, and that silencing of either one of these variants can result in destabilization of the MSCN. Ordinarily, mH2A1.1 and mH2A2 suppress Id2 expression and stabilize Snai2 in naïve MEFs, whereas their KD results in up-regulation of Id2 (which promotes the epithelial state) and suppression of Snai2, which weakens the mesenchymal phenotype. During cellular reprogramming Id2 is up-regulated and Snai2 down-regulated (Fig 2D), processes that are perfectly mimicked upon mH2A1.1 and mH2A2 KD in MEFs without the need of overexpressing OSKMs. We conclude that mH2A1.1 and mH2A2 nucleosomes (S4A Fig) directly regulate the expression of Id2 and Snai2, and by doing so they contribute to maintenance of the mesenchymal phenotype.

In addition to the critical role played by mH2A1.1 and mH2A2 in Id2 and Snai2 expression, our analysis revealed direct mH2A variant-specific effects on various MSCN genes. For example, the Fn1 gene is specifically bound and regulated by mH2A1.1 nucleosomes bound to the gene body (Figs 3 and S4B, upper panel). mH2A1.1 KD results in Fn1 down-regulation, a fact that is consistent with escape of the cells from the mesenchymal state towards reprogramming (S4B Fig). Furthermore, although Lox is bound by mH2A1.1 (upstream and gene body regions) and mH2A1.2 (upstream regions), it is down-regulated only by mH2A1.1 KD (direct effect) and mH2A2 KD (indirect effect) (S4B Fig, middle panel). Dab2 is occupied by all three mH2A variants at the gene body and at the proximal regulatory regions by mH2A1.2 and mH2A2. However, Dab2 is down-regulated only by mH2A1.2 KD (Figs 3 and S4B, middle panel). Col11a1 represents an example of a specific mH2A2 nucleosome occupation leading to gene expression changes after KD (Figs 3 and S4B, bottom panel), thus alleviating the mesenchymal phenotype. Contrary to the variant-specific effects described above, Cadm1 is negatively regulated by all mH2A variants, whereas Lamc2 is positively regulated by mH2A1.1 and negatively regulated by mH2A1.2 and mH2A2 (Fig 3 and S4C Fig). Thus, although mH2A variants can associate with the same gene, they don’t necessarily have the same role.

Furthermore, we explored the extensive binding of mH2A1.1 in ESCs and we identified many ESC-specific binding events. We found that Col4a1 and Col4a2 are occupied by mH2A1.1-bearing nucleosomes in ESCs but not in MEFs and as a result, KD of mH2A1.1 in ESCs down-regulates both genes. Similar cell-specific mH2A1.1 occupation patterns were observed in the Plaur, Lrrc15 and Htra1 genes, where mH2A1.1 depletion results in changes of their expression pattern in ESCs but not in MEFs (S4D Fig). In summary, our data show that mH2A variants directly target the majority of mH2A_MET/EMT_ genes and that the KD of individual mH2A variants affects different genes of the MSCN. This simultaneous combinatorial targeting of the 63 MSCN genes by mH2A variants can now explain why all three mH2As can block reprogramming with comparable efficiency.

## Discussion

Understanding the mechanisms that generate and maintain a variety of specialized cells, and coordinate their functions is critical for explaining how organs develop and function. The more specialized a cell is, the less developmental plasticity it acquires. Differentiated cells implement chromatin-dependent gene expression programs that safeguard their identity by generating a dense transcription factor-dependent chromatin interaction dynamic landscape, which dictates access to specific transcriptional regulatory proteins that can “read” the DNA code.

**Fig 4:**
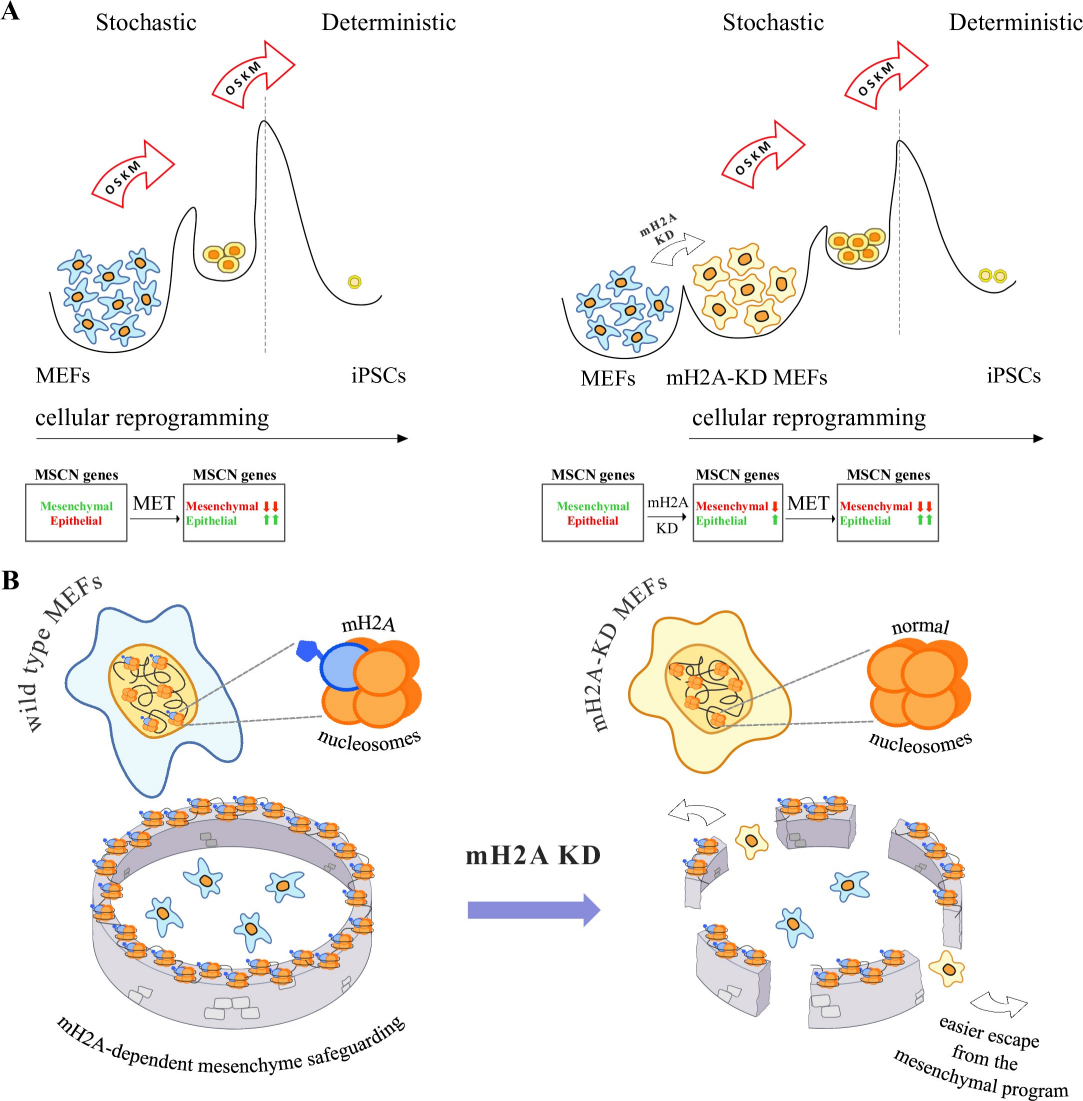
mH2A nucleosomes function as epigenetic barriers safeguarding cell identity. A. Graphic representation of a model depicting the role of mH2A nucleosomes in defining thresholds of cellular reprogramming. Left Panel: OSKM expression in MEFs induces MET via stochastic mechanisms followed by deterministic processes leading to pluripotency. In wild type MEFs (left panel), OSKM activity perturbs the steady state of the MSCN, inducing down-regulation of mesenchymal genes and up-regulation of the epithelial ones. mH2A KD induces down-regulation of the MSCN’s mesenchymal genes and up-regulation of the epithelial ones before the OSKM overexpression, lowering the barriers required for cell fate switch (Right panel). As a result, mH2A-KD MEFs can be reprogrammed more efficiently, since the cells are poised towards the epithelial state before the initiation of the process. MET during reprogramming is more efficient and, therefore, reprogramming efficiency is increased. **B.** Model describing the genomic function of mH2A-containing nucleosomes in safeguarding mesenchymal identity by stabilizing cell type-specific gene expression programs (blue cells safeguarded by mH2A nucleosomes). The presence of mH2A nucleosomes on various key mesenchyme genes acts as a barrier (depicted as a “wall”) to cellular reprogramming (left panel of Fig 4B). mH2A variants KD leads to a reduction of the number of mH2A nucleosomes throughout chromatin landscape and as a result the MEF transcription program is no longer strictly and/or robustly controlled (“wall” breaks in the figure right panel) allowing the escape to alternative cell fates.

Our study highlights the complexity of the regulatory circuits building protective mechanisms, in order to prohibit cellular reprogramming from occurring naturally (Fig 4A–4B). Ordinarily, the route to reprogramming requires, among many others, an enhanced rate of cell divisions to achieve the high division potential of embryonic stem cells, abandonment of the differentiated cell state (e.g. the mesenchymal state for fibroblasts), gaining of epithelial characteristics (MET) and metabolic drifts towards glycolysis. These global changes are coordinated by gene expression programs related to the cell cycle, cell metabolism and cell morphology [11, 39]. We showed that changes required for altering cell morphology are highly dependent on mH2A-containing nucleosomes, and as a result, depletion of any one of the three mH2A variants creates an advantageous starting point, which enables cells, like MEFs, to escape more efficiently the differentiated state towards pluripotency (Fig 4A). Therefore, loss of mesenchymal identity is a rate limiting step and mH2A nucleosomes are molecular determinants that prohibit cells to initiate transition to the epithelial state, thus preserving established gene expression programs (Fig 4B).

Paradoxically, mH2A individual variant KDs have similar effects on reprogramming. We showed that they do so by regulating the expression of different genes of the MSCN and therefore, in a way, their impact on the cell phenotype is variant-independent by targeting different nodes of the same network (Fig 4). Indeed, our network analysis strongly suggests that the concerted actions of the mH2A-regulated battery of genes can lead to protection of the differentiated cell phenotype either from induced or from accidental dedifferentiation events. mH2A nucleosomes can secure the execution of reliable gene expression programs by binding with high affinity to specific regulatory sequences and thus minimizing transcriptional noise [23]. Ordinarily, mH2A nucleosomes deposited on key genes prevent transcriptional plasticity by blocking the random access of transcription factors, which otherwise, could interfere with the accurate execution of cell type-specific gene expression programs [23]. Of note, we showed that the mesenchymal genes of the MSCN are down-regulated upon individual mH2A KDs (especially in the case of mH2A1.1 and mH2A2), an observation that agrees perfectly with the down-regulation of these genes occurring normally during cellular reprogramming. For example, in the case of the Id2/Snai2 regulatory module, the mH2A KD effects mimic the expression of Id2 and the repression of Snai2 genes, both of which occur normally during reprogramming. Therefore, we propose that the reprogramming potential of OSKM factors is significantly enhanced in mH2A KD cells since certain of the required morphological alterations can be achieved before the initiation of the process (Fig 4A).

Recent studies strongly suggest that various pathways towards carcinogenesis and cellular reprogramming intersect, thus underscoring the existence of common molecular determinants [39–41]. Both processes impose forms of “transformation” through which cells change their transcriptional and epigenetic identity and acquire altered phenotypes including immortality, unlimited proliferation potential, high telomerase activity and a metabolic switch towards glycolysis. Importantly, MET plays an indispensable role during both carcinogenesis and cellular reprogramming, but in a different manner. Cancer cells initially use EMT to extravasate and eventually MET to colonize new tissues during metastasis [42–47]. In this context, mH2A was found to suppress metastasis of cutaneous melanoma cancer cells [48], whereas, by contrast, expression of mH2A1 is linked with increased aggressiveness of uveal melanoma [49]. Similarly, mH2A1.1 inhibits EMT induction in immortalized human mammary epithelial cells [31], whereas high level expression of mH2A1 is linked with negative prognosis in metastatic breast cancer [50]. This apparent paradox of mH2A nucleosomes safeguarding opposite cancer cell fates [51], can be explained in the context of our model, where mH2A plays a role in preserving both the differentiated phenotype of MEFs and the pluripotency phenotype of ESCs by controlling the expression of distinct batteries of genes.

## Materials and methods

### Cell lines and MEFs isolation

Bruce 4 (C57/B6 Strain) mouse embryonic stem cells (ESCs) and HEK293T were purchased from ATCC. Mouse Embryonic Fibroblasts (MEFs) were isolated as previously described [8]. Briefly, E13.5 C57BL/6 mouse embryos from 13.5 day-pregnant female mice were placed in (1X) PBS, the head and embryonic internal organs were dissociated from the abdominal cavity, then trypsinized and passed through a 10-ml syringe to produce single-cell suspensions, which were further expanded. MEFs and HEK293T cells were cultured in Dulbecco’s Modified Eagle’s Medium (DMEM) Sigma-Aldrich, Cat. No. D6429) supplemented with 10% heat-inactivated Fetal Bovine Serum (FBS), Penicillin/Streptomycin (100 ug/ml; Thermo Fischer Scientific, Cat. No. 15140122), 2mM GlutaMAX (Gibco #,Cat. No. 35-050-061), and 0.1 mM Non-Essential Amino Acids 100X (Gibco, Cat. No. 11140050), at 37°C and 5% CO2. Bruce4 ESCs were cultured in complete ESC medium [Knock Out MediumDMEM (Gibco, Cat. No. 10829018), 20% ES-tested FBS (Pansera, Cat. No. P30-2602), Penicillin/Streptomycin 1X (Thermo Fischer Scientific, Cat. No. 15140122), GlutaMAX 1X (Gibco, Cat. No. 35-050-061), and Non-Essential Amino Acids 1X (Gibco, Cat. No. 11140050)], with 20 ng/mL mLIF (Santa Cruz Biotechnology, sc-4378).

### Ethics statement

This work has been approved by the Biomedical Research Foundation of Academy of Athens animal welfare and ethics committee.

### Lentivirus production, infection and shRNA knockdown (KD)

Scramble, anti-mH2A1.1, anti-mH2A1.2 and anti-mH2A2 short hairpin (sh) RNA-producing DNA sequences were cloned in PLKO.1-puro-U6 plasmids, (oligos sequences are provided in S2 Table). Reconstitution of lentiviruses was carried out in Human Embryonic Kidney 293T (HEK293T) cells by standard calcium phosphate DNA transfection protocols, using pMD2.G [a gift from Didier Trono (Addgene, Cat. No. 12259)] and psPAX2 [a gift from Didier Trono (Addgene, Cat. No. 12260)]) packaging plasmids. Lentivirus-containing supernatant was harvested 3 days after medium change.

### Transduction of MEFs and ESCs

∼500,000 of C57BL/6 early-passage MEFs (Passage 1–3) or ESCs were infected overnight with filtered viral supernatants and 8 ug/mL polybrene, followed by medium change (MEF medium, or ESC medium and recovery of the culture for an additional day). Three days post-infection, cells were selected with 5–10 μg/ml puromycin (Sigma-Aldrich, Cat. No. P8833) over a period of 7 days, followed by medium change and a two-day recovery period, after which they were harvested for downstream assays.

### MEFs cellular reprogramming—iPSCs derivation and reprogramming efficiency assays

∼400,000 MEFs bearing an OKSM polycistronic cassette under the control of a Tet-responsive element (TetO) inserted in the 3΄-UTR of Col1a1 locus (originating from the transgenic murine strain B6;129S4-*Col1a1*^*tm1(tetO*^*Col1a1*^*Tm1TetO-Pou5f1*,*-Klf4*,*-Sox2*,*-Myc)Hoch*^/J, Jackson ID, Cat. No. 011001) [52] were seeded in 10 cm plates. For the induction of OSKM expression, doxycycline was added at a 2 μg/ml final concentration. At day 7 of the reprogramming process, cells were growing in MEF media and then were switched to ESC media.

For reprogramming efficiency assays, transduced cells at the 6^th^ day of reprogramming were trypsinized to single-cell suspensions and ∼75,000 cells from each culture were seeded onto gelatin 0.1% pre-coated cell culture dishes, with Mitomycin C-treated MEFs being used as feeder layers. The cultures were maintained in mESC medium. On days 18 to 21, all cultures were stained for Alkaline Phosphatase (AP) activity, using the NBT/BCIP substrate solution (Roche Life Sciences, Cat. No. 11681451001), in NTMT buffer [100mM Tris-HCl, 100mM NaCl, 50 mM MgCl_2_ and 0.1% Tween-20, pH 9.5] and counted. Reprogramming Efficiency (RE) (%) was calculated by dividing the total number of AP-stained formations with the number of trypsinized cells seeded on day 6 on the feeder layers (∼75,000 cells) and multiplying by 100.

### Protein extraction and Western blotting analysis

Cells were lysed in RIPA lysis buffer (150 mM NaCl, 50 mM Tris-HCl pH 8.0, 1% IGEPAL, 0.5% sodium deoxycholate and 0.1% SDS) that was added with a protease inhibitor cocktail. A total protein amount of 20 ug from each sample was denatured at 95°C for 10 min in Laemmli buffer containing β-mercaptoethanol before SDS-PAGE (electrophoresis). The primary custom made antibodies anti-mH2A1.1, anti-mH2A1.2, anti-mH2A2 (SF1B-SF1E) [53], anti-B-tubulin (1:10,000) (Cell Signaling, anti-B-tubulin #2146) or anti-B-actin (Cell Signaling, anti-B-actin #3700) were diluted in PBST 1X - containing 1% skim milk and incubated at 4°C overnight. Commercial anti-mH2A1.1 (Cell Signaling Technology, Cat. No. D5F6N), anti-macroH2A1.2 (Cell Signaling Technology, Cat. No. 4287) and anti-macroH2A2 (Abcam, Cat. No. ab102126) were used as positive controls. Secondary antibodies conjugated to horseradish peroxidase (HRP) (Bio-Rad, Cat. No.1706515; Santa Cruz Biotechnology, Cat. No. sc-2055) were diluted (1:5,000) in 1X Tris-buffered saline with Tween-20 (TBST-) and membrane-incubated for 1 h at room temperature. Signals were detected with Immobilon Western chemiluminescent HRP substrate (Millipore, Cat. No. WBKLS0500).

### MNase sequencing

Isolation of mono-, di- and tri-nucleosomes, and immunoprecipitation were performed as previously described [53]. mH2A1.1, mH2A1.2 (S1B Fig) and mH2A2 affinity purified antibodies raised against variant-specific amino acid residues in rabbit [53] were used for Immunoprecipitation. DNA was purified with Agencourt AMPure beads (Beckman Coulter, Cat. No. A63881) and then subjected to library preparation for Illumina sequencing [53]. Briefly, 1–15 ng of ChIPed DNA was blunt-ended by End Repair Enzyme Mix (T4 DNA Polymerase, Klenow Fragment, T4 DNA Polynucleotide kinase), followed by “A”-tailing of 3΄-ends and ligation with the annealed TruSeq Adapters (Illumina). Conversion of the Y-shaped adapters to dsDNA occurred prior to the library size selection through 2.5% Metaphor/SeaKem LE (3:1 ratio) agarose gel electrophoresis. Each library was purified using miniElute columns (Qiagen) and then subjected to pre-amplification. The final quantification of DNA libraries was carried out according to the Quantification Standards of Illumina on an Agilent Technologies 2100 Bioanalyzer (Agilent Technologies).

### ChIP-seq analysis

The quality of the generated fastq files was examined with the FASTQC software. FASTQ files were aligned against mm10 mouse genome with Bowtie2 aligner [54]. The output file was filtered for low quality reads, duplicates, blacklist regions and mitochondrial genome entries with custom-made pipeline from Bedtools [55] and Samtools [56] packages. Peak calling was performed with SICER2 with default parameters [57]. Bigwig files were generated with BamCoverage from Deeptools with Log_2_FC (IP/Input normalization) [58]. Annotation of peaks to genes was performed with GREAT using regions ±10 kb from TSS of genes [59]. Tornado and Summary plots were constructed with computeMatrix and plotHeatmap tools from Deeptools [58]. Average binding analysis was performed with Multibigwig command from Deeptools package [58] at the selected regions after RPKM normalization.

### RNA-seq analysis

RNA-seq libraries were prepared with the Illumina TruSeq RNA v2 kit using 1 μg of total RNA, checked with the Agilent bioanalyzer DNA1000 chip, quantified with the qubit HS spectrophotometric method and pooled in equimolar amounts for sequencing. ∼25 million, 50 bp long, single-end reads were generated for each sample with Illumina NextSeq500 sequencer.

Quality Control (QC) was performed at the FASTQ raw data file for each sample using the FASTQC software. FASTQ files were aligned to mm10 mouse genome using HISAT2 [60]. Counts were defined using HTSeq htseq-count command with the “intersection non-empty” option [61]. The count files were used as input for DESeq2 [62]. Normalization was performed with the estimate size factor function followed by Differentially Expressed Genes Analysis. (DEGs) analysis. Pathway analysis was performed at EnrichR environment [63–65]. Heatmaps were constructed with pheatmap package in R, after computing the respective z-score. Data for pathway analysis of Fig 1 were retrieved from Molecular Signature Database Hallmark gene set 2020 [66]. Heatmaps were constructed with pheatmap package in R, after computing the respective z-score. Transcriptome analysis was performed with Salmon package [67].

### Network analysis

The DEGs (log_2_FC>0.58 or <-0.58, p-adjusted<0.05) from our RNA-seq analysis of mH2A1.1, mH2A1.2 and mH2A2 KD MEFs were used as input (separately) in EnrichR tool [63–65] for pathway analysis using Molecular Signature Database Hallmark gene set 2020 [66]. The genes assigned to Epithelial Mesenchymal Transition pathway from each of the three DEG sets were retrieved and incorporated in a joined gene list (73 mH2A_MET/EMT_ genes). Network analysis of the above genes was performed using STRING database [68] and Cytoscape [69], using all types of edges (physical and non-physical connections). The nodes (10 protein genes) which were not connected with the main forming network were removed from view and were not analyzed further. Expression data regarding the mH2A1.1, mH2A1.2 and mH2A2 KD MEFs from our RNA-seq analysis were integrated in the network as log_2_FC values (log_2_FC>0.58 –red color or <-0.58 –blue color, p-adjusted<0.05). Non-statistically significant alterations in gene expression were not depicted in the network (white color). The expression pattern of genes during MEF reprogramming were extracted from publicly available RNA-seq data [32] and depicted as normalized counts in the form of line charts inside the nodes. The normalized counts were estimated using DESeq2 with the estimate size factor function. The scale of the y axis is different in each line graph according to the expression value range of each gene. The predominant subcellular localization of each gene was estimated using GO cellular component data. The role of each node in the processes of MET/EMT and its relation to the epithelial/mesenchymal phenotype was determined based on scientific literature (S1 Table).

## Supporting information

S1 Fig Efficient individual KDs of mH2A variants increase reprogramming efficiency. **A.** Schematic representation of our experimental outline. KD of mH2A variants was performed in MEFs and ESCs, followed by RNA-seq and transcriptomic analysis. **B.** Western blotting analysis, validating the efficiency and specificity of the lenti-viral vectors encoding shRNAs for mH2A1.1, mH2A1.2 and mH2A2 KD using mH2A isoform-specific antibodies [53]. **C.** Bargraphs depicting normalized expression levels of mH2A1.1, mH2A1.2 and mH2A2 in wild type MEFs and ESCs as defined by RNAseq analysis. **D**. Upper panel: Bar graph summarizing the effects mH2A KDs in the reprogramming efficiency (%) of MEFs as compared to control cells expressing scramble shRNA. KD of each of the mH2As isoforms increased the efficiency of reprogramming in a statistically significant manner. Data are shown as mean ± SEM of at least three independent experiments. Pairwise comparisons between individual KDs and scramble cells were performed with two-tail Student’s t-test. Dots represent individual values (n = 3). Lower panel: Representative images of Alkaline Phosphatase assays for scramble, mH2A1.1 KD, mH2A1.2 KD and mH2A2 KD MEF cells. **E.** Western blot validating the specificity of anti-mH2A1.1 and anti-mH2A1.2 antibodies. HeLa cells were transduced with lenti-viral particles overexpressing either the mH2A1.1 or the mH2A1.2 isoform fused to a Myc tag. Anti-mH2A1.1 detects the overexpressed mH2A1.1 only and not the overexpressed mH2A1.2 and vice versa.
(TIF)

S2 Fig Variant specific effects of mH2A KDs in MEFs and ESCs. A. Venn Diagram depicting the common down-regulated DEGs in mH2A1.1 KD, mH2A1.2 KD and mH2A2 KD as compared to control MEFs (scramble). **B.** Same as in (A) except for up-regulated DEGs in MEFs. **C.** Venn Diagram depicting the common down-regulated DEGs in mH2A1.1 KD, mH2A1.2 KD and mH2A2 KD as compared to control ESCs (scramble). **D.** Same as in (C) except for up-regulated genes in ESCs. **E**. Bar graph depicting the number of DEGs affected by all three mH2A variants in MEFs and ESCs. **F.** Heatmap depicting the expression levels of the 73 mH2A_MET/EMT_ genes after isoform-specific mH2A KD in MEFs. Replicates were clustered in an unbiased manner according to the expression levels of the mH2A_MET/EMT_ genes. Color mapping refers to z-score. **G**. Same as in (F) except that the analysis was performed in ESCs. In contrast to the mH2A2 KD effects in MEFs (7 genes), depletion of mH2A2 in ESCs led to the up-regulation of a significantly larger number of genes (33 genes).
(TIF)

S3 Fig ChIP-seq analysis revealing the mH2A1.1, mH2A1.2 and mH2A2 binding patterns in MEFs and ESCs. A. Summary and Tornado plots depicting the binding of mH2A1.1 (left panels), mH2A1.2 (middle panels) and mH2A2 (right panels), in MEFs (upper panels) and ESCs (lower panels). Signal is normalized as log_2_FC (IP signal/Input signal) and peaks were defined using SICER2. **B.** Venn diagrams depicting the mH2A individual variant targets of the 73 mH2A_MET/EMT_ genes in MEFs and ESCs. Targets were defined using the broad peaks derived from peak-calling analysis with SICER2 and peaks were annotated to genes with GREAT tool (±10 kb from the TSS). mH2A1.1 and mH2A2 have the most targets in MEFs, whereas in ESCs mH2A1.1 is the primary variant with direct binding at the 73 mH2A_MET/EMT_ gene loci. **C.** Heatmaps depicting comparative ChIPseq analysis of mH2A1.1, mH2A1.2 and mH2A2 variants bound to the 73 mH2A_MET/EMT_ genes in MEFs and ESCs as indicated. The average mH2A binding was calculated either at the -5kb regulatory region upstream from TSS or at the gene bodies after RPKM normalization. **D.** Intersection of the data presented in Fig 2B and S3C Fig. Genes with direct binding of a mH2A-bearing nucleosome are depicted in yellow and genes with no significant mH2A binding are depicted in black.
(TIF)

S4 Fig Examples of variant and cell type-specific binding of mH2A nucleosomes at the 73 mH2A_MET/EMT_ genes. Related to Fig 3. A. Genome viewer snapshots of mH2A nucleosomes at the proximal regulatory regions of Id2 and Snai2. The effect of the respective KD is depicted at the right side of the tracks. Red bars depict statistically significant peaks as defined by Sicer2. Open rectangle depicts mH2A nucleosomes with putative regulatory roles. Signals are calculated as log_2_FC (IP signal/ Input signal). Id2 and Snai2 expression is affected by mH2A1.1 and mH2A2 KD, in agreement with the statistically significant binding. B. Same as in (A) except for the Fn1, Lox, Dab2 and Col11a1 genes. C. Same as in (A) except for the Cadm1 and Lamc1. D. Genome viewer snapshots of mH2A nucleosomes at the proximal regulatory regions of of the indicated genes in MEFs and ESCs state.
(TIF)

S1 Table The role of the 73 mH2A_MET/EMT_ genes in the regulation and acquisition of an epithelial, or mesenchymal-like phenotype. Related to Fig 2. In this table, each of the 73 mH2A_MET/EMT_ genes has been assigned to a role regarding the regulation of the epithelial, or the mesenchymal phenotype and the processes of MET and EMT based on thorough bibliographic inspection. The fill color of each table cell depicts the role assigned to the corresponding gene like in Fig 2D (orange: genes related exclusively, or mostly to EMT/mesenchymal phenotype, green: genes related exclusively, or mostly to MET/epithelial phenotype and grey: genes related to both EMT/mesenchymal and MET/epithelial phenotypes depending on the context, or with inconclusive evidence).
(DOCX)

S2 Table Short-hairpin (sh) RNA producing DNA sequences used for every individual mH2A variant KD. Related to S1 Fig.
(DOCX)
