# Supplementary figures and images for: Combinatorial targeting of a specific EMT/MET network by macroH2A variants safeguards mesenchymal identity
Source: PLoS One. 2023 Jul 11;18(7):e0288005. doi: 10.1371/journal.pone.0288005 (PMC10335705; doi:10.1371/journal.pone.0288005)

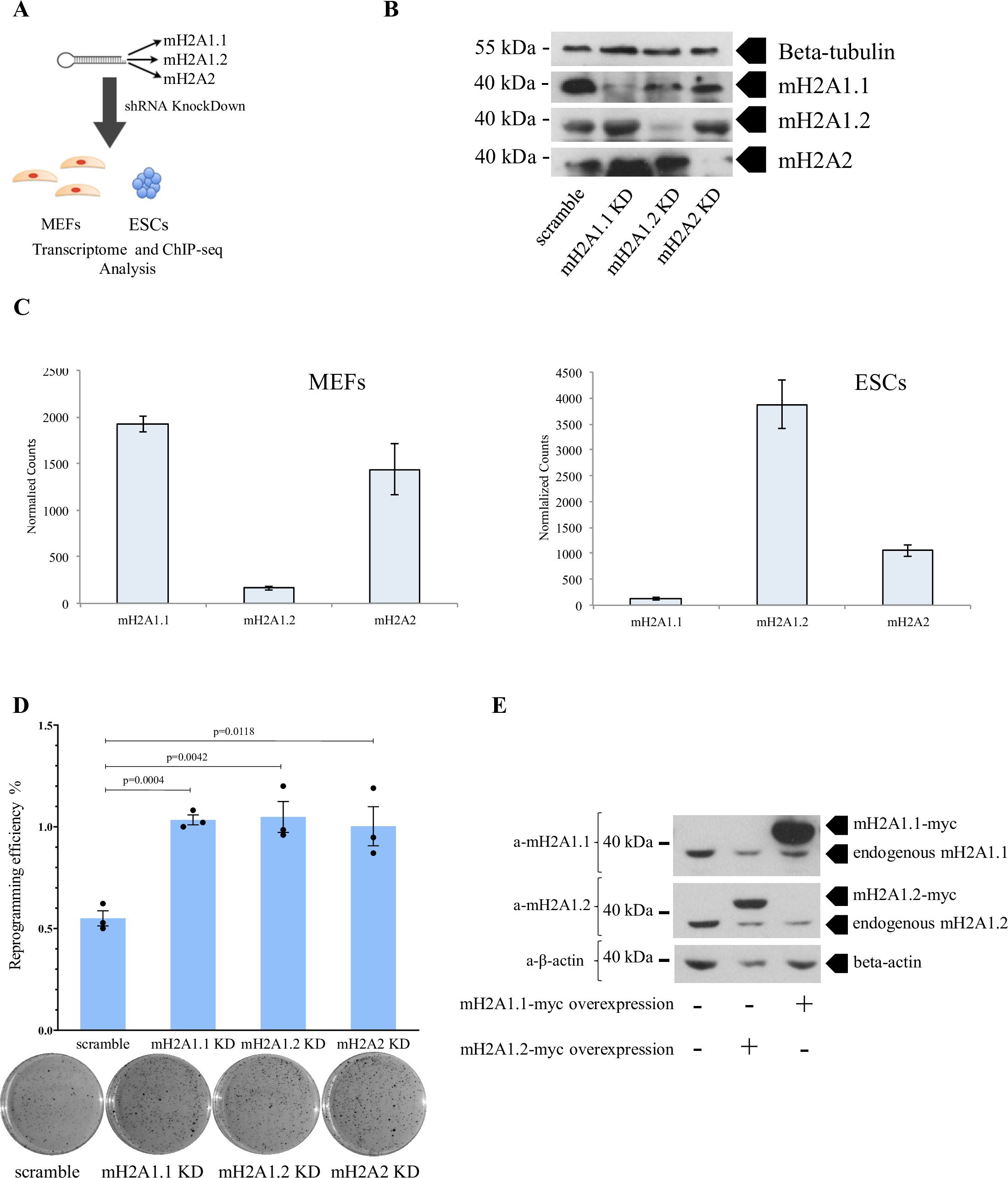

Supplement: S1 Fig — A. Schematic representation of our experimental outline. KD of mH2A variants was performed in MEFs and ESCs, followed by RNA-seq and transcriptomic analysis. B. Western blotting analysis, validating the efficiency and specificity of the lenti-viral vectors encoding shRNAs for mH2A1.1, mH2A1.2 and mH2A2 KD using mH2A isoform-specific antibodies [53]. C. Bargraphs depicting normalized expression levels of mH2A1.1, mH2A1.2 and mH2A2 in wild type MEFs and ESCs as defined by RNAseq analysis. D. Upper panel: Bar graph summarizing the effects mH2A KDs in the reprogramming efficiency (%) of MEFs as compared to control cells expressing scramble shRNA. KD of each of the mH2As isoforms increased the efficiency of reprogramming in a statistically significant manner. Data are shown as mean ± SEM of at least three independent experiments. Pairwise comparisons between individual KDs and scramble cells were performed with two-tail Student’s t-test. Dots represent individual values (n = 3). Lower panel: Representative images of Alkaline Phosphatase assays for scramble, mH2A1.1 KD, mH2A1.2 KD and mH2A2 KD MEF cells. E. Western blot validating the specificity of anti-mH2A1.1 and anti-mH2A1.2 antibodies. HeLa cells were transduced with lenti-viral particles overexpressing either the mH2A1.1 or the mH2A1.2 isoform fused to a Myc tag. Anti-mH2A1.1 detects the overexpressed mH2A1.1 only and not the overexpressed mH2A1.2 and vice versa. (TIF) [file pone.0288005.s001.tif]

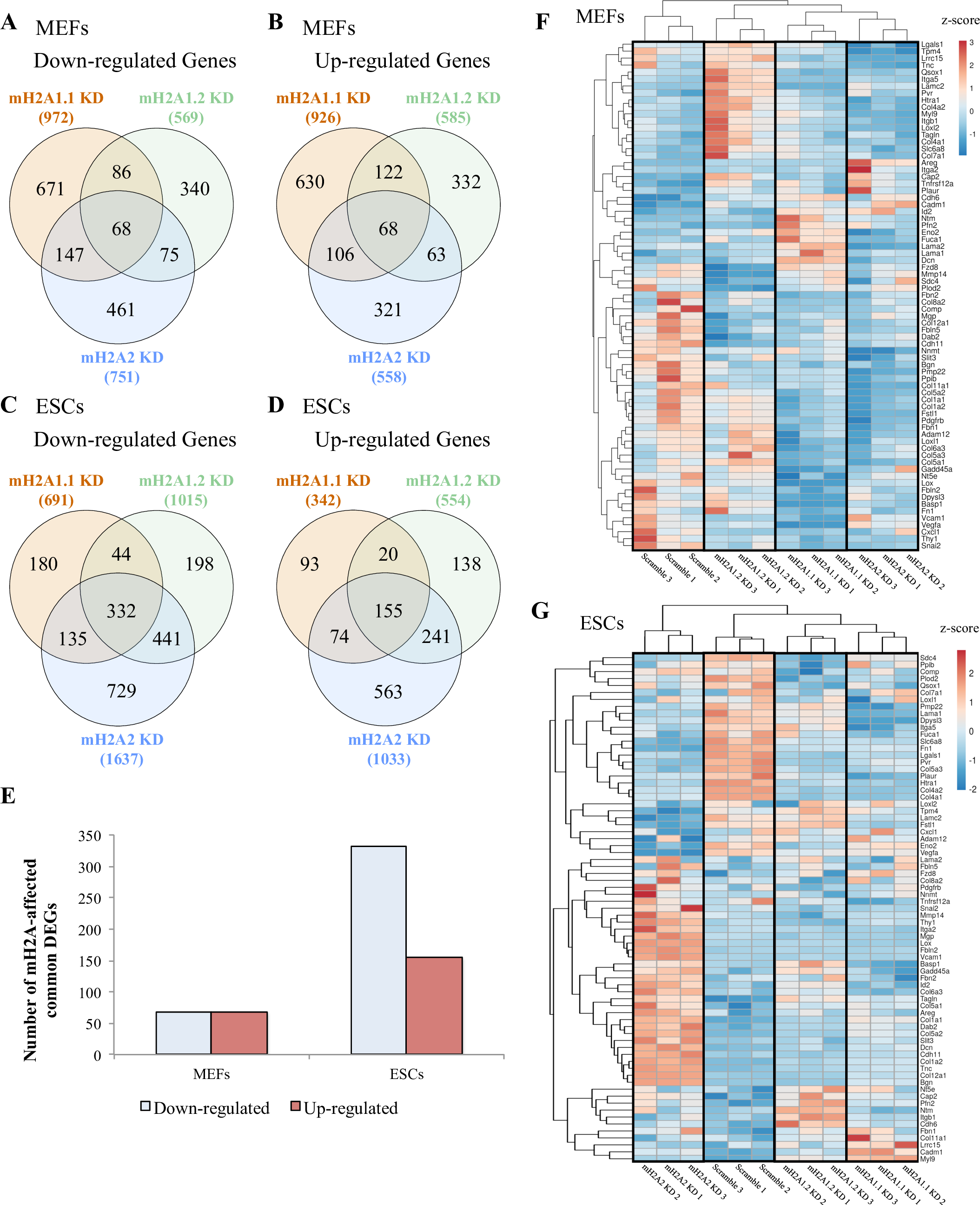

Supplement: S2 Fig — Venn Diagram depicting the common down-regulated DEGs in mH2A1.1 KD, mH2A1.2 KD and mH2A2 KD as compared to control MEFs (scramble). B. Same as in (A) except for up-regulated DEGs in MEFs. C. Venn Diagram depicting the common down-regulated DEGs in mH2A1.1 KD, mH2A1.2 KD and mH2A2 KD as compared to control ESCs (scramble). D. Same as in (C) except for up-regulated genes in ESCs. E. Bar graph depicting the number of DEGs affected by all three mH2A variants in MEFs and ESCs. F. Heatmap depicting the expression levels of the 73 mH2AMET/EMT genes after isoform-specific mH2A KD in MEFs. Replicates were clustered in an unbiased manner according to the expression levels of the mH2AMET/EMT genes. Color mapping refers to z-score. G. Same as in (F) except that the analysis was performed in ESCs. In contrast to the mH2A2 KD effects in MEFs (7 genes), depletion of mH2A2 in ESCs led to the up-regulation of a significantly larger number of genes (33 genes). (TIF) [file pone.0288005.s002.tif]

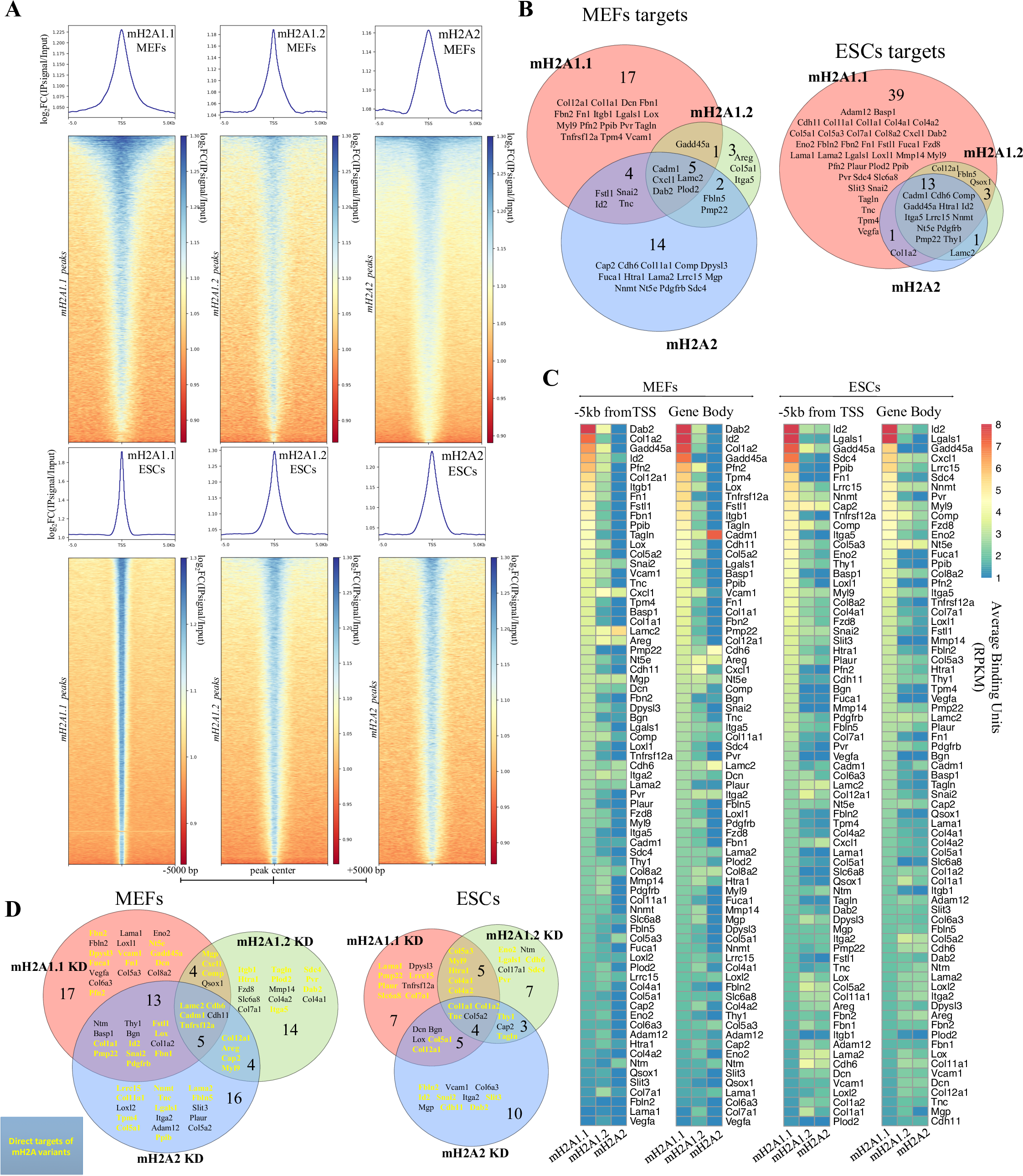

Supplement: S3 Fig — Summary and Tornado plots depicting the binding of mH2A1.1 (left panels), mH2A1.2 (middle panels) and mH2A2 (right panels), in MEFs (upper panels) and ESCs (lower panels). Signal is normalized as log2FC (IP signal/Input signal) and peaks were defined using SICER2. B. Venn diagrams depicting the mH2A individual variant targets of the 73 mH2AMET/EMT genes in MEFs and ESCs. Targets were defined using the broad peaks derived from peak-calling analysis with SICER2 and peaks were annotated to genes with GREAT tool (±10 kb from the TSS). mH2A1.1 and mH2A2 have the most targets in MEFs, whereas in ESCs mH2A1.1 is the primary variant with direct binding at the 73 mH2AMET/EMT gene loci. C. Heatmaps depicting comparative ChIPseq analysis of mH2A1.1, mH2A1.2 and mH2A2 variants bound to the 73 mH2AMET/EMT genes in MEFs and ESCs as indicated. The average mH2A binding was calculated either at the -5kb regulatory region upstream from TSS or at the gene bodies after RPKM normalization. D. Intersection of the data presented in Fig 2B and S3C Fig. Genes with direct binding of a mH2A-bearing nucleosome are depicted in yellow and genes with no significant mH2A binding are depicted in black. (TIF) [file pone.0288005.s003.tif]

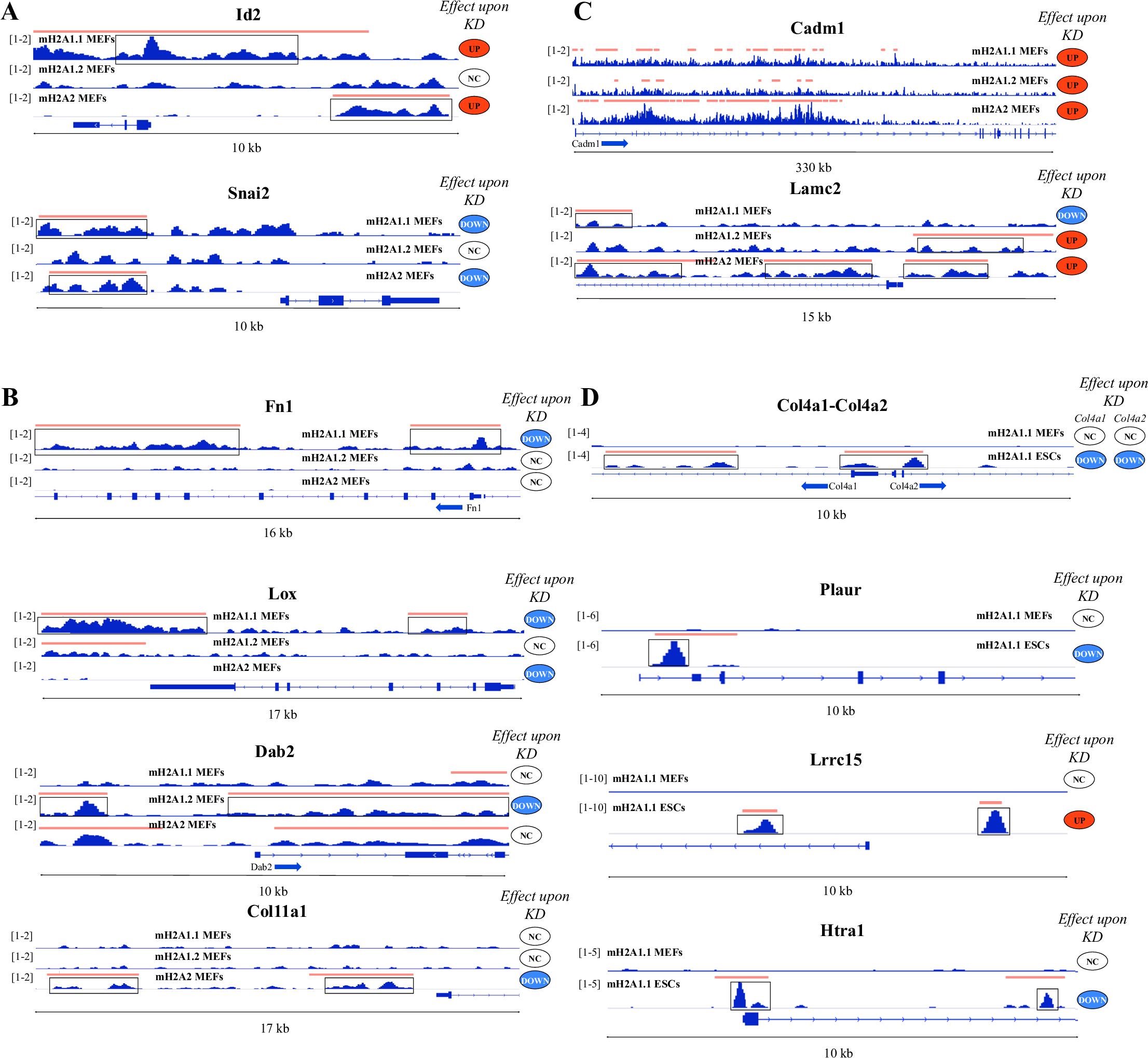

Supplement: S4 Fig — Related to Fig 3. A. Genome viewer snapshots of mH2A nucleosomes at the proximal regulatory regions of Id2 and Snai2. The effect of the respective KD is depicted at the right side of the tracks. Red bars depict statistically significant peaks as defined by Sicer2. Open rectangle depicts mH2A nucleosomes with putative regulatory roles. Signals are calculated as log2FC (IP signal/ Input signal). Id2 and Snai2 expression is affected by mH2A1.1 and mH2A2 KD, in agreement with the statistically significant binding. B. Same as in (A) except for the Fn1, Lox, Dab2 and Col11a1 genes. C. Same as in (A) except for the Cadm1 and Lamc1. D. Genome viewer snapshots of mH2A nucleosomes at the proximal regulatory regions of of the indicated genes in MEFs and ESCs state. (TIF) [file pone.0288005.s004.tif]
