# Supplementary material for: Combinatorial targeting of a specific EMT/MET network by macroH2A variants safeguards mesenchymal identity
Source: PLoS One. 2023 Jul 11;18(7):e0288005. doi: 10.1371/journal.pone.0288005 (PMC10335705; doi:10.1371/journal.pone.0288005)
Supplement: S1 Table — Related to Fig 2. In this table, each of the 73 mH2AMET/EMT genes has been assigned to a role regarding the regulation of the epithelial, or the mesenchymal phenotype and the processes of MET and EMT based on thorough bibliographic inspection. The fill color of each table cell depicts the role assigned to the corresponding gene like in Fig 2D (orange: genes related exclusively, or mostly to EMT/mesenchymal phenotype, green: genes related exclusively, or mostly to MET/epithelial phenotype and grey: genes related to both EMT/mesenchymal and MET/epithelial phenotypes depending on the context, or with inconclusive evidence). (DOCX) [file pone.0288005.s005.docx]

The fill color of each table cell depicts the role assigned to the corresponding gene in MET or EMT; **orange**: genes related exclusively, or mostly to EMT/mesenchymal phenotype, **green**: genes related exclusively, or mostly to MET/epithelial phenotype, and **grey**: genes related to both EMT/mesenchymal and MET/epithelial phenotypes depending on the context, or with inconclusive evidence.

| mH2A_MET-EMT_ genes | **Related with** | **EMT/mesenchymal phenotype references** | **MET/epithelial phenotype references** |
| --- | --- | --- | --- |
| **Adam12** | EMT/mesenchymal phenotype | [1–4] |  |
| **Areg** | both EMT/mesenchymal phenotype and MET/epithelial phenotype, or inconclusive evidence | [5,6] | [5,6] |
| **Basp1** | both EMT/mesenchymal phenotype and MET/epithelial phenotype, or inconclusive evidence | [7] | [8] |
| **Bgn** | EMT/mesenchymal phenotype | [9–11] |  |
| **Cadm1** | MET/epithelial phenotype |  | [12]. [13] |
| **Cap2** | EMT/mesenchymal phenotype | [14–16] |  |
| **Cdh6** | both EMT/mesenchymal phenotype and MET/epithelial phenotype, or inconclusive evidence | [17–21] | [22,23] |
| **Cdh11** | mostly EMT/mesenchymal phenotype | [24–27] | [28,29]. |
| **Col1a1** | EMT/mesenchymal phenotype | [30–36] |  |
| **Col1a2** | mostly EMT/mesenchymal phenotype | [30,32,33,37,38] | [39] |
| **Col4a1** | both EMT/mesenchymal phenotype and MET/epithelial phenotype, or inconclusive evidence | [32,35,40–43] | [31,34] |
| **Col4a2** | both EMT/mesenchymal phenotype and MET/epithelial phenotype, or inconclusive evidence | [32,35,43] | [31] |
| **Col5a1** | EMT/mesenchymal phenotype | [30,44,45] |  |
| **Col5a2** | EMT/mesenchymal phenotype | [30,32,46] |  |
| **Col5a3** | EMT/mesenchymal phenotype | [30] |  |
| **Col6a3** | EMT/mesenchymal phenotype | [47] |  |
| **Col7a1** | both EMT/mesenchymal phenotype and MET/epithelial phenotype, or inconclusive evidence | [48,49] | [50,51] |
| **Col8a2** | EMT/mesenchymal phenotype | [52–54] |  |
| **Col11a1** | EMT/mesenchymal phenotype | [55–59] |  |
| **Col12a1** | EMT/mesenchymal phenotype | [60,61] |  |
| **Comp** | EMT/mesenchymal phenotype | [62,63] |  |
| **Cxcl1** | EMT/mesenchymal phenotype | [64–70] |  |
| **Dab2** | both EMT/mesenchymal phenotype and MET/epithelial phenotype, or inconclusive evidence | [71] | [72] |
| **Dcn** | MET/epithelial phenotype |  | [73–75] |
| **Dpysl3** | both EMT/mesenchymal phenotype and MET/epithelial phenotype, or inconclusive evidence | [76,77] | [78,79]. |
| **Eno2** | EMT/mesenchymal phenotype | [80–82] |  |
| **Fbln2** | both EMT/mesenchymal phenotype and MET/epithelial phenotype, or inconclusive evidence | [83] | [83] |
| **Fbln5** | both EMT/mesenchymal phenotype and MET/epithelial phenotype, or inconclusive evidence | [83,84] | [83] |
| **Fbn1** | both EMT/mesenchymal phenotype and MET/epithelial phenotype, or inconclusive evidence | [85,86] | [87,88] |
| **Fbn2** | EMT/mesenchymal phenotype | [86,88–90] |  |
| **Fn1** | EMT/mesenchymal phenotype | [91–94] |  |
| **Fstl1** | EMT/mesenchymal phenotype | [95–98] |  |
| **Fuca1** | MET/epithelial phenotype |  | [99–102] |
| **Fzd8** | EMT/mesenchymal phenotype | [103–105] |  |
| **Gadd45a** | MET/epithelial phenotype |  | [106–108] |
| **Htra1** | MET/epithelial phenotype |  | [109–113] |
| **Id2** | mostly MET/epithelial phenotype | [114,115] | [116–118] |
| **Itga2** | both EMT/mesenchymal phenotype and MET/epithelial phenotype, or inconclusive evidence | [119,120] | [121,122] |
| **Itga5** | EMT/mesenchymal phenotype | [123–125] |  |
| **Itgb1** | EMT/mesenchymal phenotype | [92,126–129] |  |
| **Lama1** | EMT/mesenchymal phenotype | [130] |  |
| **Lama2** | MET/epithelial phenotype |  | [31,131] |
| **Lamc2** | EMT/mesenchymal phenotype | [132–138] |  |
| **Lgals1** | EMT/mesenchymal phenotype | [139–142] |  |
| **Lox** | EMT/mesenchymal phenotype | [143–146] |  |
| **Loxl1** | EMT/mesenchymal phenotype | [147] |  |
| **Loxl2** | EMT/mesenchymal phenotype | [148,149] |  |
| **Lrrc15** | EMT/mesenchymal phenotype | [150,151] |  |
| **Mgp** | mostly EMT/mesenchymal phenotype | [152–156] | [157,158] |
| **Mmp14** | EMT/mesenchymal phenotype | [159–162] |  |
| **Myl9** | EMT/mesenchymal phenotype | [163,164] |  |
| **Nnmt** | EMT/mesenchymal phenotype | [165–168] |  |
| **Nt5e** | EMT/mesenchymal phenotype | [169–172] |  |
| **Ntm** | mostly EMT/mesenchymal phenotype | [173,174] | [175] |
| **Pdgfrb** | EMT/mesenchymal phenotype | [176] |  |
| **Pfn2** | mostly EMT/mesenchymal phenotype | [177–182] | [183] |
| **Plaur** | mostly EMT/mesenchymal phenotype | [184–186] | [187] |
| **Plod2** | EMT/mesenchymal phenotype | [188–190] |  |
| **Pmp22** | mostly EMT/mesenchymal phenotype | [191–194] | [195] |
| **Ppib** | EMT/mesenchymal phenotype | [196–201] |  |
| **Pvr** | mostly EMT/mesenchymal phenotype | [202–208] | [209] |
| **Qsox1** | EMT/mesenchymal phenotype | [210–213] |  |
| **Sdc4** | both EMT/mesenchymal phenotype and MET/epithelial phenotype, or inconclusive evidence | [214,215] | [216,217] |
| **Slc6a8** | both EMT/mesenchymal phenotype and MET/epithelial phenotype, or inconclusive evidence | [218,219] | [220] |
| **Slit3** | mostly MET/epithelial phenotype | [221] | [222–224] |
| **Snai2** | EMT/mesenchymal phenotype | [225,226] |  |
| **Tagln** | both EMT/mesenchymal phenotype and MET/epithelial phenotype, or inconclusive evidence | [227–235] | [227,228,236–240] |
| **Thy1** | EMT/mesenchymal phenotype | [241] |  |
| **Tnc** | EMT/mesenchymal phenotype | [242,243] |  |
| **Tnfrsf12a** | EMT/mesenchymal phenotype | [94,244–251] |  |
| **Tpm4** | both EMT/mesenchymal phenotype and MET/epithelial phenotype, or inconclusive evidence | [252] | [253,254] |
| **Vegfa** | mostly EMT/mesenchymal phenotype | [255,256] | [257] |
| **Vcam1** | mostly EMT/mesenchymal phenotype | [258–263] | [264] |

**References**

1. Ruff M, Leyme A, Le Cann F, Bonnier D, Le Seyec J, Chesnel F, et al. The Disintegrin and Metalloprotease ADAM12 Is Associated with TGF-β-Induced Epithelial to Mesenchymal Transition. Katoh M, editor. PLoS One. 2015;10: e0139179. doi:10.1371/journal.pone.0139179

2. Duhachek-Muggy S, Qi Y, Wise R, Alyahya L, Li H, Hodge J, et al. Metalloprotease-disintegrin ADAM12 actively promotes the stem cell-like phenotype in claudin-low breast cancer. Mol Cancer. 2017;16: 32. doi:10.1186/s12943-017-0599-6

3. Eckert MA, Santiago-Medina M, Lwin TM, Kim J, Courtneidge SA, Yang J. ADAM12 induction by TWIST1 promotes tumor invasion and metastasis via regulation of invadopodia and focal adhesions. J Cell Sci. 2017;130: 2036–2048. doi:10.1242/jcs.198200

4. Iba K, Albrechtsen R, Gilpin B, Fröhlich C, Loechel F, Zolkiewska A, et al. The Cysteine-Rich Domain of Human Adam 12 Supports Cell Adhesion through Syndecans and Triggers Signaling Events That Lead to β1 Integrin–Dependent Cell Spreading. J Cell Biol. 2000;149: 1143–1156. doi:10.1083/jcb.149.5.1143

5. Berasain C, Avila MA. Amphiregulin. Semin Cell Dev Biol. 2014;28: 31–41. doi:10.1016/j.semcdb.2014.01.005

6. Busser B, Sancey L, Brambilla E, Coll J-L, Hurbin A. The multiple roles of amphiregulin in human cancer. Biochim Biophys Acta - Rev Cancer. 2011;1816: 119–131. doi:10.1016/j.bbcan.2011.05.003

7. Wang X, Cao Y, BoPan B, Meng Q, Yu Y. High BASP1 Expression is Associated with Poor Prognosis and Promotes Tumor Progression in Human Lung Adenocarcinoma. Cancer Invest. 2021;39: 409–422. doi:10.1080/07357907.2021.1910290

8. Xu W, Ji J, Xu Y, Liu Y, Shi L, Liu Y, et al. MicroRNA-191, by promoting the EMT and increasing CSC-like properties, is involved in neoplastic and metastatic properties of transformed human bronchial epithelial cells. Mol Carcinog. 2015;54: E148–E161. doi:10.1002/mc.22221

9. Li H, Zhong A, Li S, Meng X, Wang X, Xu F, et al. The integrated pathway of TGFβ/Snail with TNFα/NFκB may facilitate the tumor-stroma interaction in the EMT process and colorectal cancer prognosis. Sci Rep. 2017;7: 4915. doi:10.1038/s41598-017-05280-6

10. Schulz GB, Grimm T, Sers C, Riemer P, Elmasry M, Kirchner T, et al. Prognostic value and association with epithelial-mesenchymal transition and molecular subtypes of the proteoglycan biglycan in advanced bladder cancer. Urol Oncol Semin Orig Investig. 2019;37: 530.e9-530.e18. doi:10.1016/j.urolonc.2019.05.011

11. Hu L, Duan Y, Li J, Su L, Yan M, Zhu Z, et al. Biglycan enhances gastric cancer invasion by activating FAK signaling pathway. Oncotarget. 2014;5: 1885–1896. doi:10.18632/oncotarget.1871

12. Hartsough EJ, Weiss MB, Heilman SA, Purwin TJ, Kugel CH, Rosenbaum SR, et al. CADM1 is a TWIST1-regulated suppressor of invasion and survival. Cell Death Dis. 2019;10: 281. doi:10.1038/s41419-019-1515-3

13. Sakurai-Yageta M, Masuda M, Tsuboi Y, Ito A, Murakami Y. Tumor suppressor CADM1 is involved in epithelial cell structure. Biochem Biophys Res Commun. 2009;390: 977–982. doi:10.1016/j.bbrc.2009.10.088

14. Shi Y, Li Z, Zhou Z, Liao S, Wu Z, Li J, et al. Identification and validation of an epithelial mesenchymal transition-related gene pairs signature for prediction of overall survival in patients with skin cutaneous melanoma. PeerJ. 2022;10: e12646. doi:10.7717/peerj.12646

15. Liang F, Wang R, Du Q, Zhu S. An Epithelial–Mesenchymal Transition Hallmark Gene-Based Risk Score System in Head and Neck Squamous-Cell Carcinoma. Int J Gen Med. 2021;Volume 14: 4219–4227. doi:10.2147/IJGM.S327632

16. Masugi Y, Tanese K, Emoto K, Yamazaki K, Effendi K, Funakoshi T, et al. Overexpression of adenylate cyclase-associated protein 2 is a novel prognostic marker in malignant melanoma. Pathol Int. 2015;65: 627–634. doi:10.1111/pin.12351

17. Sancisi V, Gandolfi G, Ragazzi M, Nicoli D, Tamagnini I, Piana S, et al. Cadherin 6 Is a New RUNX2 Target in TGF-β Signalling Pathway. Li J, editor. PLoS One. 2013;8: e75489. doi:10.1371/journal.pone.0075489

18. Gugnoni M, Sancisi V, Gandolfi G, Manzotti G, Ragazzi M, Giordano D, et al. Cadherin-6 promotes EMT and cancer metastasis by restraining autophagy. Oncogene. 2017;36: 667–677. doi:10.1038/onc.2016.237

19. Clay MR, Halloran MC. Cadherin 6 promotes neural crest cell detachment via F-actin regulation and influences active Rho distribution during epithelial-to-mesenchymal transition. Development. 2014;141: 2506–2515. doi:10.1242/dev.105551

20. Park K-S, Gumbiner BM. Cadherin-6B stimulates an epithelial mesenchymal transition and the delamination of cells from the neural ectoderm via LIMK/cofilin mediated non-canonical BMP receptor signaling. Dev Biol. 2012;366: 232–243. doi:10.1016/j.ydbio.2012.04.005

21. Park K-S, Gumbiner BM. Cadherin 6B induces BMP signaling and de-epithelialization during the epithelial mesenchymal transition of the neural crest. Development. 2010;137: 2691–2701. doi:10.1242/dev.050096

22. Taneyhill LA, Coles EG, Bronner-Fraser M. Snail2 directly represses cadherin6B during epithelial-to-mesenchymal transitions of the neural crest. Development. 2007;134: 1481–1490. doi:10.1242/dev.02834

23. Coles EG, Taneyhill LA, Bronner-Fraser M. A critical role for Cadherin6B in regulating avian neural crest emigration. Dev Biol. 2007;312: 533–544. doi:10.1016/j.ydbio.2007.09.056

24. Alimperti S, Andreadis ST. CDH2 and CDH11 act as regulators of stem cell fate decisions. Stem Cell Res. 2015;14: 270–282. doi:10.1016/j.scr.2015.02.002

25. Chen J-H, Huang W-C, Bamodu OA, Chang PM-H, Chao T-Y, Huang T-H. Monospecific antibody targeting of CDH11 inhibits epithelial-to-mesenchymal transition and represses cancer stem cell-like phenotype by up-regulating miR-335 in metastatic breast cancer, in vitro and in vivo. BMC Cancer. 2019;19: 634. doi:10.1186/s12885-019-5811-1

26. Kim N-H, Choi S-H, Lee TR, Lee C-H, Lee A-Y. Cadherin 11, a miR-675 Target, Induces N-Cadherin Expression and Epithelial–Mesenchymal Transition in Melasma. J Invest Dermatol. 2014;134: 2967–2976. doi:10.1038/jid.2014.257

27. Schneider DJ, Wu M, Le TT, Cho S, Brenner MB, Blackburn MR, et al. Cadherin‐11 contributes to pulmonary fibrosis: potential role in TGF‐β production and epithelial to mesenchymal transition. FASEB J. 2012;26: 503–512. doi:10.1096/fj.11-186098

28. Li L, Ying J, Li H, Zhang Y, Shu X, Fan Y, et al. The human cadherin 11 is a pro-apoptotic tumor suppressor modulating cell stemness through Wnt/β-catenin signaling and silenced in common carcinomas. Oncogene. 2012;31: 3901–3912. doi:10.1038/onc.2011.541

29. Piao S, Inglehart RC, Scanlon CS, Russo N, Banerjee R, D’Silva NJ. CDH11 inhibits proliferation and invasion in head and neck cancer. J Oral Pathol Med. 2017;46: 89–97. doi:10.1111/jop.12471

30. Xu S, Xu H, Wang W, Li S, Li H, Li T, et al. The role of collagen in cancer: from bench to bedside. J Transl Med. 2019;17: 309. doi:10.1186/s12967-019-2058-1

31. Banerjee S, Lo W-C, Majumder P, Roy D, Ghorai M, Shaikh NK, et al. Multiple roles for basement membrane proteins in cancer progression and EMT. Eur J Cell Biol. 2022;101: 151220. doi:10.1016/j.ejcb.2022.151220

32. Yin W, Zhu H, Tan J, Xin Z, Zhou Q, Cao Y, et al. Identification of collagen genes related to immune infiltration and epithelial-mesenchymal transition in glioma. Cancer Cell Int. 2021;21: 276. doi:10.1186/s12935-021-01982-0

33. Medici D, Nawshad A. Type I collagen promotes epithelial–mesenchymal transition through ILK-dependent activation of NF-κB and LEF-1. Matrix Biol. 2010;29: 161–165. doi:10.1016/j.matbio.2009.12.003

34. Zeisberg M, Neilson EG. Biomarkers for epithelial-mesenchymal transitions. J Clin Invest. 2009;119: 1429–1437. doi:10.1172/JCI36183

35. Reka AK, Chen G, Jones RC, Amunugama R, Kim S, Karnovsky A, et al. Epithelial-mesenchymal transition-associated secretory phenotype predicts survival in lung cancer patients. Carcinogenesis. 2014;35: 1292–1300. doi:10.1093/carcin/bgu041

36. Li X, Sun X, Kan C, Chen B, Qu N, Hou N, et al. COL1A1: A novel oncogenic gene and therapeutic target in malignancies. Pathol - Res Pract. 2022;236: 154013. doi:10.1016/j.prp.2022.154013

37. Ponticos M, Harvey C, Ikeda T, Abraham D, Bou-Gharios G. JunB mediates enhancer/promoter activity of COL1A2 following TGF-β induction. Nucleic Acids Res. 2009;37: 5378–5389. doi:10.1093/nar/gkp544

38. Tamilzhalagan S, Rathinam D, Ganesan K. Amplified 7q21-22 gene MCM7 and its intronic miR-25 suppress COL1A2 associated genes to sustain intestinal gastric cancer features. Mol Carcinog. 2017;56: 1590–1602. doi:10.1002/mc.22614

39. Yu Y, Liu D, Liu Z, Li S, Ge Y, Sun W, et al. The inhibitory effects of COL1A2 on colorectal cancer cell proliferation, migration, and invasion. J Cancer. 2018;9: 2953–2962. doi:10.7150/jca.25542

40. Zhang H, Wang Y, Ding H. COL4A1, negatively regulated by XPD and miR-29a-3p, promotes cell proliferation, migration, invasion and epithelial–mesenchymal transition in liver cancer cells. Clin Transl Oncol. 2021;23: 2078–2089. doi:10.1007/s12094-021-02611-y

41. Hu Y-Z, Hu Z-L, Liao T-Y, Li Y, Pan Y-L. LncRNA SND1-IT1 facilitates TGF-β1-induced epithelial-to-mesenchymal transition via miR-124/COL4A1 axis in gastric cancer. Cell Death Discov. 2022;8: 73. doi:10.1038/s41420-021-00793-6

42. Chen S-Y, Lin J-S, Yang B-C. Modulation of tumor cell stiffness and migration by type IV collagen through direct activation of integrin signaling pathway. Arch Biochem Biophys. 2014;555–556: 1–8. doi:10.1016/j.abb.2014.05.004

43. Jechlinger M, Grunert S, Tamir IH, Janda E, Lüdemann S, Waerner T, et al. Expression profiling of epithelial plasticity in tumor progression. Oncogene. 2003;22: 7155–7169. doi:10.1038/sj.onc.1206887

44. Lei G-S, Kline HL, Lee C-H, Wilkes DS, Zhang C. Regulation of Collagen V Expression and Epithelial-Mesenchymal Transition by miR-185 and miR-186 during Idiopathic Pulmonary Fibrosis. Am J Pathol. 2016;186: 2310–2316. doi:10.1016/j.ajpath.2016.04.015

45. Park Y, Park M, Kim J, Ahn J, Sim J, Bang J-I, et al. NOX2-Induced High Glycolytic Activity Contributes to the Gain of COL5A1-Mediated Mesenchymal Phenotype in GBM. Cancers (Basel). 2022;14: 516. doi:10.3390/cancers14030516

46. Sun J, Han S, Chen P. LncRNA XIST contributes to epithelial–mesenchymal transformation in posterior opacity via regulating miR-98-5p/COL5A2 axis and PI3K/Akt/FOXO1 pathway. Mol Cell Toxicol. 2022. doi:10.1007/s13273-022-00247-9

47. Chen P, Cescon M, Bonaldo P. Collagen VI in cancer and its biological mechanisms. Trends Mol Med. 2013;19: 410–417. doi:10.1016/j.molmed.2013.04.001

48. König A, Bruckner-Tuderman L. Transforming growth factor-beta stimulates collagen VII expression by cutaneous cells in vitro. J Cell Biol. 1992;117: 679–685. doi:10.1083/jcb.117.3.679

49. Ortiz-Urda S, Garcia J, Green CL, Chen L, Lin Q, Veitch DP, et al. Type VII Collagen Is Required for Ras-Driven Human Epidermal Tumorigenesis. Science (80- ). 2005;307: 1773–1776. doi:10.1126/science.1106209

50. Nyström A, Velati D, Mittapalli VR, Fritsch A, Kern JS, Bruckner-Tuderman L. Collagen VII plays a dual role in wound healing. J Clin Invest. 2013;123: 3498–3509. doi:10.1172/JCI68127

51. Martins VL, Vyas JJ, Chen M, Purdie K, Mein CA, South AP, et al. Increased invasive behaviour in cutaneous squamous cell carcinoma with loss of basement-membrane type VII collagen. J Cell Sci. 2009;122: 1788–1799. doi:10.1242/jcs.042895

52. Hou G, Mulholland D, Gronska MA, Bendeck MP. Type VIII Collagen Stimulates Smooth Muscle Cell Migration and Matrix Metalloproteinase Synthesis after Arterial Injury. Am J Pathol. 2000;156: 467–476. doi:10.1016/S0002-9440(10)64751-7

53. Skrbic B, Engebretsen KVT, Strand ME, Lunde IG, Herum KM, Marstein HS, et al. Lack of collagen VIII reduces fibrosis and promotes early mortality and cardiac dilatation in pressure overload in mice†. Cardiovasc Res. 2015;106: 32–42. doi:10.1093/cvr/cvv041

54. Loeffler I, Liebisch M, Wolf G. Collagen VIII influences epithelial phenotypic changes in experimental diabetic nephropathy. Am J Physiol Physiol. 2012;303: F733–F745. doi:10.1152/ajprenal.00212.2012

55. Sok JC, Lee JA, Dasari S, Joyce S, Contrucci SC, Egloff AM, et al. Collagen type XI α1 facilitates head and neck squamous cell cancer growth and invasion. Br J Cancer. 2013;109: 3049–3056. doi:10.1038/bjc.2013.624

56. Freire J, Domínguez-Hormaetxe S, Pereda S, De Juan A, Vega A, Simón L, et al. Collagen, type XI, alpha 1: An accurate marker for differential diagnosis of breast carcinoma invasiveness in core needle biopsies. Pathol - Res Pract. 2014;210: 879–884. doi:10.1016/j.prp.2014.07.012

57. Wu Y-H, Chou C-Y. Collagen XI Alpha 1 Chain, a Novel Therapeutic Target for Cancer Treatment. Front Oncol. 2022;12: 1–9. doi:10.3389/fonc.2022.925165

58. Raglow Z, Thomas SM. Tumor matrix protein collagen XIα1 in cancer. Cancer Lett. 2015;357: 448–453. doi:10.1016/j.canlet.2014.12.011

59. Nallanthighal S, Heiserman JP, Cheon D-J. Collagen Type XI Alpha 1 (COL11A1): A Novel Biomarker and a Key Player in Cancer. Cancers (Basel). 2021;13: 935. doi:10.3390/cancers13050935

60. Xiang Z, Li J, Song S, Wang J, Cai W, Hu W, et al. A positive feedback between IDO1 metabolite and COL12A1 via MAPK pathway to promote gastric cancer metastasis. J Exp Clin Cancer Res. 2019;38: 314. doi:10.1186/s13046-019-1318-5

61. Jiang X, Wu M, Xu X, Zhang L, Huang Y, Xu Z, et al. COL12A1, a novel potential prognostic factor and therapeutic target in gastric cancer. Mol Med Rep. 2019;20: 3103–3112. doi:10.3892/mmr.2019.10548

62. Nfonsam VN, Nfonsam LE, Chen D, Omesiete PN, Cruz A, Runyan RB, et al. COMP Gene Coexpresses With EMT Genes and Is Associated With Poor Survival in Colon Cancer Patients. J Surg Res. 2019;233: 297–303. doi:10.1016/j.jss.2018.08.021

63. Zhong W, Hou H, Liu T, Su S, Xi X, Liao Y, et al. Cartilage Oligomeric Matrix Protein promotes epithelial-mesenchymal transition by interacting with Transgelin in Colorectal Cancer. Theranostics. 2020;10: 8790–8806. doi:10.7150/thno.44456

64. Cheng W-L, Wang C-S, Huang Y-H, Tsai M-M, Liang Y, Lin K-H. Overexpression of CXCL1 and its receptor CXCR2 promote tumor invasion in gastric cancer. Ann Oncol. 2011;22: 2267–2276. doi:10.1093/annonc/mdq739

65. Kuo P-L, Shen K-H, Hung S-H, Hsu Y-L. CXCL1/GROα increases cell migration and invasion of prostate cancer by decreasing fibulin-1 expression through NF-κB/HDAC1 epigenetic regulation. Carcinogenesis. 2012;33: 2477–2487. doi:10.1093/carcin/bgs299

66. Hsu Y-L, Chen Y-J, Chang W-A, Jian S-F, Fan H-L, Wang J-Y, et al. Interaction between Tumor-Associated Dendritic Cells and Colon Cancer Cells Contributes to Tumor Progression via CXCL1. Int J Mol Sci. 2018;19: 2427. doi:10.3390/ijms19082427

67. Wang N, Liu W, Zheng Y, Wang S, Yang B, Li M, et al. CXCL1 derived from tumor-associated macrophages promotes breast cancer metastasis via activating NF-κB/SOX4 signaling. Cell Death Dis. 2018;9: 880. doi:10.1038/s41419-018-0876-3

68. Lu Y, Dong B, Xu F, Xu Y, Pan J, Song J, et al. CXCL1-LCN2 paracrine axis promotes progression of prostate cancer via the Src activation and epithelial-mesenchymal transition. Cell Commun Signal. 2019;17: 118. doi:10.1186/s12964-019-0434-3

69. Alafate W, Li X, Zuo J, Zhang H, Xiang J, Wu W, et al. Elevation of CXCL1 indicates poor prognosis and radioresistance by inducing mesenchymal transition in glioblastoma. CNS Neurosci Ther. 2020;26: 475–485. doi:10.1111/cns.13297

70. Korbecki J, Barczak K, Gutowska I, Chlubek D, Baranowska-Bosiacka I. CXCL1: Gene, Promoter, Regulation of Expression, mRNA Stability, Regulation of Activity in the Intercellular Space. Int J Mol Sci. 2022;23: 792. doi:10.3390/ijms23020792

71. Prunier C, Howe PH. Disabled-2 (Dab2) Is Required for Transforming Growth Factor β-induced Epithelial to Mesenchymal Transition (EMT). J Biol Chem. 2005;280: 17540–17548. doi:10.1074/jbc.M500974200

72. Martin JC, Herbert B-S, Hocevar BA. Disabled-2 downregulation promotes epithelial-to-mesenchymal transition. Br J Cancer. 2010;103: 1716–1723. doi:10.1038/sj.bjc.6605975

73. Mao L, Yang J, Yue J, Chen Y, Zhou H, Fan D, et al. Decorin deficiency promotes epithelial-mesenchymal transition and colon cancer metastasis. Matrix Biol. 2021;95: 1–14. doi:10.1016/j.matbio.2020.10.001

74. Bi X, Pohl NM, Qian Z, Yang GR, Gou Y, Guzman G, et al. Decorin-mediated inhibition of colorectal cancer growth and migration is associated with E-cadherin in vitro and in mice. Carcinogenesis. 2012;33: 326–330. doi:10.1093/carcin/bgr293

75. Hu X, Villodre ES, Larson R, Rahal OM, Wang X, Gong Y, et al. Decorin-mediated suppression of tumorigenesis, invasion, and metastasis in inflammatory breast cancer. Commun Biol. 2021;4: 72. doi:10.1038/s42003-020-01590-0

76. Matsunuma R, Chan DW, Kim B-J, Singh P, Han A, Saltzman AB, et al. DPYSL3 modulates mitosis, migration, and epithelial-to-mesenchymal transition in claudin-low breast cancer. Proc Natl Acad Sci. 2018;115: E11978–E11987. doi:10.1073/pnas.1810598115

77. Hiroshima Y, Nakamura F, Miyamoto H, Mori R, Taniguchi K, Matsuyama R, et al. Collapsin Response Mediator Protein 4 Expression is Associated with Liver Metastasis and Poor Survival in Pancreatic Cancer. Ann Surg Oncol. 2013;20: 369–378. doi:10.1245/s10434-012-2491-3

78. Gao X, Pang J, Li L-Y, Liu W-P, Di J-M, Sun Q-P, et al. Expression profiling identifies new function of collapsin response mediator protein 4 as a metastasis-suppressor in prostate cancer. Oncogene. 2010;29: 4555–4566. doi:10.1038/onc.2010.213

79. ZHOU W, XIE P, PANG M, YANG B, FANG Y, SHU T, et al. Upregulation of CRMP4, a new prostate cancer metastasis suppressor gene, inhibits tumor growth in a nude mouse intratibial injection model. Int J Oncol. 2015;46: 290–298. doi:10.3892/ijo.2014.2705

80. Tang C, Wang M, Dai Y, Wei X. Krüppel-like factor 12 suppresses bladder cancer growth through transcriptionally inhibition of enolase 2. Gene. 2021;769: 145338. doi:10.1016/j.gene.2020.145338

81. Zheng Y, Wu C, Yang J, Zhao Y, Jia H, Xue M, et al. Insulin-like growth factor 1-induced enolase 2 deacetylation by HDAC3 promotes metastasis of pancreatic cancer. Signal Transduct Target Ther. 2020;5: 53. doi:10.1038/s41392-020-0146-6

82. Lu L, Zha Z, Zhang P, Li D, Liu G. NSE, positively regulated by LINC00657-miR-93-5p axis, promotes small cell lung cancer (SCLC) invasion and epithelial-mesenchymal transition (EMT) process. Int J Med Sci. 2021;18: 3768–3779. doi:10.7150/ijms.58415

83. Mahajan D, Kancharla S, Kolli P, Sharma AK, Singh S, Kumar S, et al. Role of Fibulins in Embryonic Stage Development and Their Involvement in Various Diseases. Biomolecules. 2021;11: 685. doi:10.3390/biom11050685

84. Lee Y-H, Albig AR, Regner M, Schiemann BJ, Schiemann WP. Fibulin-5 initiates epithelial-mesenchymal transition (EMT) and enhances EMT induced by TGF- in mammary epithelial cells via a MMP-dependent mechanism. Carcinogenesis. 2008;29: 2243–2251. doi:10.1093/carcin/bgn199

85. Li L, Liao J, Yuan Q, Hong X, Li J, Peng Y, et al. Fibrillin-1–enriched microenvironment drives endothelial injury and vascular rarefaction in chronic kidney disease. Sci Adv. 2021;7: 1–14. doi:10.1126/sciadv.abc7170

86. Kiemer AK, Takeuchi K, Quinlan MP. Identification of genes involved in epithelial-mesenchymal transition and tumor progression. Oncogene. 2001;20: 6679–6688. doi:10.1038/sj.onc.1204872

87. Peng D, Fu M, Wang M, Wei Y, Wei X. Targeting TGF-β signal transduction for fibrosis and cancer therapy. Mol Cancer. 2022;21: 104. doi:10.1186/s12943-022-01569-x

88. van Loon K, Yemelyanenko-Lyalenko J, Margadant C, Griffioen AW, Huijbers EJM. Role of fibrillin-2 in the control of TGF-β activation in tumor angiogenesis and connective tissue disorders. Biochim Biophys Acta - Rev Cancer. 2020;1873: 188354. doi:10.1016/j.bbcan.2020.188354

89. Brinckmann J, Hunzelmann N, Kahle B, Rohwedel J, Kramer J, Gibson MA, et al. Enhanced fibrillin-2 expression is a general feature of wound healing and sclerosis: potential alteration of cell attachment and storage of TGF-β. Lab Investig. 2010;90: 739–752. doi:10.1038/labinvest.2010.49

90. Boyer AS, Erickson CP, Runyan RB. Epithelial-mesenchymal transformation in the embryonic heart is mediated through distinct pertussis toxin-sensitive and TGF? signal transduction mechanisms. Dev Dyn. 1999;214: 81–91. doi:10.1002/(SICI)1097-0177(199901)214:1<81::AID-DVDY8>3.0.CO;2-3

91. Brown MS, Muller KE, Pattabiraman DR. Quantifying the Epithelial-to-Mesenchymal Transition (EMT) from Bench to Bedside. Cancers (Basel). 2022;14: 1138. doi:10.3390/cancers14051138

92. Ding Y, Pan Y, Liu S, Jiang F, Jiao J. Elevation of MiR-9–3p suppresses the epithelial-mesenchymal transition of nasopharyngeal carcinoma cells via down-regulating FN1, ITGB1 and ITGAV. Cancer Biol Ther. 2017;18: 414–424. doi:10.1080/15384047.2017.1323585

93. Griggs LA, Hassan NT, Malik RS, Griffin BP, Martinez BA, Elmore LW, et al. Fibronectin fibrils regulate TGF-β1-induced Epithelial-Mesenchymal Transition. Matrix Biol. 2017;60–61: 157–175. doi:10.1016/j.matbio.2017.01.001

94. Chen G, Sun J, Xie M, Yu S, Tang Q, Chen L. PLAU Promotes Cell Proliferation and Epithelial-Mesenchymal Transition in Head and Neck Squamous Cell Carcinoma. Front Genet. 2021;12: 1–14. doi:10.3389/fgene.2021.651882

95. Liu T, Liu Y, Miller M, Cao L, Zhao J, Wu J, et al. Autophagy plays a role in FSTL1-induced epithelial mesenchymal transition and airway remodeling in asthma. Am J Physiol Cell Mol Physiol. 2017;313: L27–L40. doi:10.1152/ajplung.00510.2016

96. Lau MC-C, Ng KY, Wong TL, Tong M, Lee TK, Ming X-Y, et al. FSTL1 Promotes Metastasis and Chemoresistance in Esophageal Squamous Cell Carcinoma through NFκB–BMP Signaling Cross-talk. Cancer Res. 2017;77: 5886–5899. doi:10.1158/0008-5472.CAN-17-1411

97. Sundaram GM, Quah S, Guang LG, Sampath P. HuR enhances FSTL1 transcript stability to promote invasion and metastasis of squamous cell carcinoma. Am J Cancer Res. 2021;11: 4981–4993. Available: http://www.ncbi.nlm.nih.gov/pubmed/34765305%0Ahttp://www.pubmedcentral.nih.gov/articlerender.fcgi?artid=PMC8569354

98. Wu M, Ding Y, Wu N, Jiang J, Huang Y, Zhang F, et al. FSTL1 promotes growth and metastasis in gastric cancer by activating AKT related pathway and predicts poor survival. Am J Cancer Res. 2021;11: 712–728. Available: http://www.ncbi.nlm.nih.gov/pubmed/33791149%0Ahttp://www.pubmedcentral.nih.gov/articlerender.fcgi?artid=PMC7994156

99. Yuan K, Listinsky CM, Singh RK, Listinsky JJ, Siegal GP. Cell Surface Associated Alpha-l-Fucose Moieties Modulate Human Breast Cancer Neoplastic Progression. Pathol Oncol Res. 2008;14: 145–156. doi:10.1007/s12253-008-9036-x

100. Cheng T-C, Tu S-H, Chen L-C, Chen M-Y, Chen W-Y, Lin Y-K, et al. Down-regulation of α-L-fucosidase 1 expression confers inferior survival for triple-negative breast cancer patients by modulating the glycosylation status of the tumor cell surface. Oncotarget. 2015;6: 21283–21300. doi:10.18632/oncotarget.4238

101. Vecchio G, Parascandolo A, Allocca C, Ugolini C, Basolo F, Moracci M, et al. Human α-L-fucosidase-1 attenuates the invasive properties of thyroid cancer. Oncotarget. 2017;8: 27075–27092. doi:10.18632/oncotarget.15635

102. Guo J, Li X, Tan Z, Lu W, Yang G, Guan F. Alteration of N-glycans and Expression of Their Related Glycogenes in the Epithelial-Mesenchymal Transition of HCV29 Bladder Epithelial Cells. Molecules. 2014;19: 20073–20090. doi:10.3390/molecules191220073

103. Yang Q, Wang Y, Pan X, Ye J, Gan S, Qu F, et al. Frizzled 8 promotes the cell proliferation and metastasis of renal cell carcinoma. Oncotarget. 2017;8: 78989–79002. doi:10.18632/oncotarget.20742

104. Li Q, Ye L, Zhang X, Wang M, Lin C, Huang S, et al. FZD8, a target of p53, promotes bone metastasis in prostate cancer by activating canonical Wnt/β-catenin signaling. Cancer Lett. 2017;402: 166–176. doi:10.1016/j.canlet.2017.05.029

105. Murillo-Garzón V, Gorroño-Etxebarria I, Åkerfelt M, Puustinen MC, Sistonen L, Nees M, et al. Frizzled-8 integrates Wnt-11 and transforming growth factor-β signaling in prostate cancer. Nat Commun. 2018;9: 1747. doi:10.1038/s41467-018-04042-w

106. Chen K, Long Q, Wang T, Zhao D, Zhou Y, Qi J, et al. Gadd45a is a heterochromatin relaxer that enhances <scp>iPS</scp> cell generation. EMBO Rep. 2016;17: 1641–1656. doi:10.15252/embr.201642402

107. Li L, Chen K, Wu Y, Long Q, Zhao D, Ma B, et al. Gadd45a opens up the promoter regions of miR-295 facilitating pluripotency induction. Cell Death Dis. 2017;8: e3107–e3107. doi:10.1038/cddis.2017.497

108. Hong L, Sun Q-F, Xu T-Y, Wu Y-H, Zhang H, Fu R-Q, et al. New role and molecular mechanism of Gadd45a in hepatic ﬁbrosis. World J Gastroenterol. 2016;22: 2779. doi:10.3748/wjg.v22.i9.2779

109. Wang N, Eckert KA, Zomorrodi AR, Xin P, Pan W, Shearer DA, et al. Down-Regulation of HtrA1 Activates the Epithelial-Mesenchymal Transition and ATM DNA Damage Response Pathways. Batra SK, editor. PLoS One. 2012;7: e39446. doi:10.1371/journal.pone.0039446

110. Zhu F, Duan Y-F, Bao W-Y, Liu W-S, Yang Y, Cai H-H. HtrA1 regulates epithelial–mesenchymal transition in hepatocellular carcinoma. Biochem Biophys Res Commun. 2015;467: 589–594. doi:10.1016/j.bbrc.2015.09.105

111. Oka C, Tsujimoto R, Kajikawa M, Koshiba-Takeuchi K, Ina J, Yano M, et al. HtrA1 serine protease inhibits signaling mediated by Tgfβ family proteins. Development. 2004;131: 1041–1053. doi:10.1242/dev.00999

112. Graham JR, Chamberland A, Lin Q, Li XJ, Dai D, Zeng W, et al. Serine Protease HTRA1 Antagonizes Transforming Growth Factor-β Signaling by Cleaving Its Receptors and Loss of HTRA1 In Vivo Enhances Bone Formation. Malaval L, editor. PLoS One. 2013;8: e74094. doi:10.1371/journal.pone.0074094

113. Pei X, Ma K, Xu J, Wang N, Liu N. Inhibition of cell proliferation and migration after HTRA1 knockdown in retinal pigment epithelial cells. Graefe’s Arch Clin Exp Ophthalmol. 2015;253: 565–572. doi:10.1007/s00417-014-2901-2

114. KAMATA Y, SUMIDA T, KOBAYASHI Y, ISHIKAWA A, KUMAMARU W, MORI Y. Introduction of ID2 Enhances Invasiveness in ID2-null Oral Squamous Cell Carcinoma Cells via the SNAIL Axis. Cancer Genomics Proteomics. 2016;13: 493–498. doi:10.21873/cgp.20012

115. Zeng S, Zhang Y, Ma J, Deng G, Qu Y, Guo C, et al. BMP4 promotes metastasis of hepatocellular carcinoma by an induction of epithelial–mesenchymal transition via upregulating ID2. Cancer Lett. 2017;390: 67–76. doi:10.1016/j.canlet.2016.12.042

116. Chang C, Yang X, Pursell B, Mercurio AM. Id2 Complexes with the SNAG Domain of Snai1 Inhibiting Snai1-Mediated Repression of Integrin β4. Mol Cell Biol. 2013;33: 3795–3804. doi:10.1128/mcb.00434-13

117. Wen XF, Chen M, Wu Y, Chen MN, Glogowska A, Klonisch T, et al. Inhibitor of DNA Binding 2 Inhibits Epithelial-Mesenchymal Transition via Up-Regulation of Notch3 in Breast Cancer. Transl Oncol. 2018;11: 1259–1270. doi:10.1016/j.tranon.2018.07.015

118. Moustakas A, Heldin P. TGFβ and matrix-regulated epithelial to mesenchymal transition. Biochim Biophys Acta - Gen Subj. 2014;1840: 2621–2634. doi:10.1016/j.bbagen.2014.02.004

119. Chuang Y-C, Wu H-Y, Lin Y-L, Tzou S-C, Chuang C-H, Jian T-Y, et al. Blockade of ITGA2 Induces Apoptosis and Inhibits Cell Migration in Gastric Cancer. Biol Proced Online. 2018;20: 10. doi:10.1186/s12575-018-0073-x

120. Wang Q, Cao T, Guo K, Zhou Y, Liu H, Pan Y, et al. Regulation of Integrin Subunit Alpha 2 by miR-135b-5p Modulates Chemoresistance in Gastric Cancer. Front Oncol. 2020;10: 1–12. doi:10.3389/fonc.2020.00308

121. Ferraro A, Boni T, Pintzas A. EZH2 Regulates Cofilin Activity and Colon Cancer Cell Migration by Targeting ITGA2 Gene. Hoheisel JD, editor. PLoS One. 2014;9: e115276. doi:10.1371/journal.pone.0115276

122. Ding W, Fan X-L, Xu X, Huang J-Z, Xu S-H, Geng Q, et al. Epigenetic Silencing of ITGA2 by MiR-373 Promotes Cell Migration in Breast Cancer. Guan X-Y, editor. PLoS One. 2015;10: e0135128. doi:10.1371/journal.pone.0135128

123. Chen J, Ji T, Wu D, Jiang S, Zhao J, Lin H, et al. Human mesenchymal stem cells promote tumor growth via MAPK pathway and metastasis by epithelial mesenchymal transition and integrin α5 in hepatocellular carcinoma. Cell Death Dis. 2019;10: 425. doi:10.1038/s41419-019-1622-1

124. Deng Y, Wan Q, Yan W. Integrin α5/ITGA5 Promotes The Proliferation, Migration, Invasion And Progression Of Oral Squamous Carcinoma By Epithelial–Mesenchymal Transition. Cancer Manag Res. 2019;Volume 11: 9609–9620. doi:10.2147/CMAR.S223201

125. Lei Y, Yan W, Lin Z, Liu J, Tian D, Han P. Comprehensive analysis of partial epithelial mesenchymal transition‐related genes in hepatocellular carcinoma. J Cell Mol Med. 2021;25: 448–462. doi:10.1111/jcmm.16099

126. Yang J, Hou Y, Zhou M, Wen S, Zhou J, Xu L, et al. Twist induces epithelial-mesenchymal transition and cell motility in breast cancer via ITGB1-FAK/ILK signaling axis and its associated downstream network. Int J Biochem Cell Biol. 2016;71: 62–71. doi:10.1016/j.biocel.2015.12.004

127. Zhang Y-Y, Kong L-Q, Zhu X-D, Cai H, Wang C-H, Shi W-K, et al. CD31 regulates metastasis by inducing epithelial–mesenchymal transition in hepatocellular carcinoma via the ITGB1-FAK-Akt signaling pathway. Cancer Lett. 2018;429: 29–40. doi:10.1016/j.canlet.2018.05.004

128. Ren L, Mo W, Wang L, Wang X. Matrine suppresses breast cancer metastasis by targeting ITGB1 and inhibiting epithelial‑to‑mesenchymal transition. Exp Ther Med. 2019; 367–374. doi:10.3892/etm.2019.8207

129. Guo D, Zhang D, Ren M, Lu G, Zhang X, He S, et al. THBS4 promotes HCC progression by regulating ITGB1 via FAK/PI3K/AKT pathway. FASEB J. 2020;34: 10668–10681. doi:10.1096/fj.202000043R

130. Yang H, Huo P, Hu G, Wei B, Kong D, Li H. Identification of gene markers associated with metastasis in clear cell renal cell carcinoma. Oncol Lett. 2017;13: 4755–4761. doi:10.3892/ol.2017.6084

131. Liang J, Li H, Han J, Jiang J, Wang J, Li Y, et al. Mex3a interacts with LAMA2 to promote lung adenocarcinoma metastasis via PI3K/AKT pathway. Cell Death Dis. 2020;11: 614. doi:10.1038/s41419-020-02858-3

132. Hintermann E, Quaranta V. Epithelial cell motility on laminin-5: regulation by matrix assembly, proteolysis, integrins and erbB receptors. Matrix Biol. 2004;23: 75–85. doi:10.1016/j.matbio.2004.03.001

133. Koshikawa N, Giannelli G, Cirulli V, Miyazaki K, Quaranta V. Role of Cell Surface Metalloprotease Mt1-Mmp in Epithelial Cell Migration over Laminin-5. J Cell Biol. 2000;148: 615–624. doi:10.1083/jcb.148.3.615

134. Moon YW, Rao G, Kim JJ, Shim H-S, Park K-S, An SS, et al. LAMC2 enhances the metastatic potential of lung adenocarcinoma. Cell Death Differ. 2015;22: 1341–1352. doi:10.1038/cdd.2014.228

135. Pei Y-F, Liu J, Cheng J, Wu W-D, Liu X-Q. Silencing of LAMC2 Reverses Epithelial-Mesenchymal Transition and Inhibits Angiogenesis in Cholangiocarcinoma via Inactivation of the Epidermal Growth Factor Receptor Signaling Pathway. Am J Pathol. 2019;189: 1637–1653. doi:10.1016/j.ajpath.2019.03.012

136. Cen W, Li J, Tong C, Zhang W, Zhao Y, Lu B, et al. Intrahepatic Cholangiocarcinoma Cells Promote Epithelial-mesenchymal Transition of Hepatocellular Carcinoma Cells by Secreting LAMC2. J Cancer. 2021;12: 3448–3457. doi:10.7150/jca.55627

137. Okada Y, Takahashi N, Takayama T, Goel A. LAMC2 promotes cancer progression and gemcitabine resistance through modulation of EMT and ATP-binding cassette transporters in pancreatic ductal adenocarcinoma. Carcinogenesis. 2021;42: 546–556. doi:10.1093/carcin/bgab011

138. Simon-Assmann P, Spenle C, Lefebvre O, Kedinger M. The Role of the Basement Membrane as a Modulator of Intestinal Epithelial–Mesenchymal Interactions. Progress in Molecular Biology and Translational Science. Elsevier Inc.; 2010. pp. 175–206. doi:10.1016/B978-0-12-381280-3.00008-7

139. Bacigalupo ML, Manzi M, Espelt M V., Gentilini LD, Compagno D, Laderach DJ, et al. Galectin-1 Triggers Epithelial-Mesenchymal Transition in Human Hepatocellular Carcinoma Cells. J Cell Physiol. 2015;230: 1298–1309. doi:10.1002/jcp.24865

140. Chong Y, Tang D, Gao J, Jiang X, Xu C, Xiong Q, et al. Galectin-1 induces invasion and the epithelial-mesenchymal transition in human gastric cancer cells via non-canonical activation of the hedgehog signaling pathway. Oncotarget. 2016;7: 83611–83626. doi:10.18632/oncotarget.13201

141. Wu D, Kanda A, Liu Y, Kase S, Noda K, Ishida S. Galectin‐1 promotes choroidal neovascularization and subretinal fibrosis mediated via epithelialmesenchymal transition. FASEB J. 2019;33: 2498–2513. doi:10.1096/fj.201801227R

142. You X, Wu J, Zhao X, Jiang X, Tao W, Chen Z, et al. Fibroblastic galectin-1-fostered invasion and metastasis are mediated by TGF-β1-induced epithelial-mesenchymal transition in gastric cancer. Aging (Albany NY). 2021;13: 18464–18481. doi:10.18632/aging.203295

143. Zhao L, Niu H, Liu Y, Wang L, Zhang N, Zhang G, et al. LOX inhibition downregulates MMP-2 and MMP-9 in gastric cancer tissues and cells. J Cancer. 2019;10: 6481–6490. doi:10.7150/jca.33223

144. Schietke R, Warnecke C, Wacker I, Schödel J, Mole DR, Campean V, et al. The Lysyl Oxidases LOX and LOXL2 Are Necessary and Sufficient to Repress E-cadherin in Hypoxia. J Biol Chem. 2010;285: 6658–6669. doi:10.1074/jbc.M109.042424

145. Wang T-H, Hsia S-M, Shieh T-M. Lysyl Oxidase and the Tumor Microenvironment. Int J Mol Sci. 2016;18: 62. doi:10.3390/ijms18010062

146. Baker A-M, Bird D, Welti JC, Gourlaouen M, Lang G, Murray GI, et al. Lysyl Oxidase Plays a Critical Role in Endothelial Cell Stimulation to Drive Tumor Angiogenesis. Cancer Res. 2013;73: 583–594. doi:10.1158/0008-5472.CAN-12-2447

147. Hu Q, Masuda T, Kuramitsu S, Tobo T, Sato K, Kidogami S, et al. Potential association of LOXL1 with peritoneal dissemination in gastric cancer possibly via promotion of EMT. Najbauer J, editor. PLoS One. 2020;15: e0241140. doi:10.1371/journal.pone.0241140

148. Park P-G, Jo SJ, Kim MJ, Kim HJ, Lee JH, Park CK, et al. Role of LOXL2 in the epithelial-mesenchymal transition and colorectal cancer metastasis. Oncotarget. 2017;8: 80325–80335. doi:10.18632/oncotarget.18170

149. Zhang Q, Yang L, Guan G, Cheng P, Cheng W, Wu A. LOXL2 Upregulation in Gliomas Drives Tumorigenicity by Activating Autophagy to Promote TMZ Resistance and Trigger EMT. Front Oncol. 2020;10: 1–17. doi:10.3389/fonc.2020.569584

150. Purcell JW, Tanlimco SG, Hickson J, Fox M, Sho M, Durkin L, et al. LRRC15 Is a Novel Mesenchymal Protein and Stromal Target for Antibody–Drug Conjugates. Cancer Res. 2018;78: 4059–4072. doi:10.1158/0008-5472.CAN-18-0327

151. Ray U, Pathoulas CL, Thirusangu P, Purcell JW, Kannan N, Shridhar V. Exploiting LRRC15 as a Novel Therapeutic Target in Cancer. Cancer Res. 2022;82: 1675–1681. doi:10.1158/0008-5472.CAN-21-3734

152. Mertsch S, Schurgers LJ, Weber K, Paulus W, Senner V. Matrix gla protein (MGP): an overexpressed and migration-promoting mesenchymal component in glioblastoma. BMC Cancer. 2009;9: 302. doi:10.1186/1471-2407-9-302

153. Fu M-H, Wang C-Y, Hsieh Y-T, Fang K-M, Tzeng S-F. Functional Role of Matrix gla Protein in Glioma Cell Migration. Mol Neurobiol. 2018;55: 4624–4636. doi:10.1007/s12035-017-0677-1

154. Wang M, Chen L, Chen Y, Wei R, Guo Q, Zhu S, et al. Intracellular matrix Gla protein promotes tumor progression by activating JAK2/STAT5 signaling in gastric cancer. Mol Oncol. 2020;14: 1045–1058. doi:10.1002/1878-0261.12652

155. Huang C, Wang M, Wang J, Wu D, Gao Y, Huang K, et al. Suppression MGP inhibits tumor proliferation and reverses oxaliplatin resistance in colorectal cancer. Biochem Pharmacol. 2021;189: 114390. doi:10.1016/j.bcp.2020.114390

156. Gong C, Zou J, Zhang M, Zhang J, Xu S, Zhu S, et al. Upregulation of MGP by HOXC8 promotes the proliferation, migration, and EMT processes of triple‐negative breast cancer. Mol Carcinog. 2019;58: 1863–1875. doi:10.1002/mc.23079

157. Tiago DM, Conceição N, Caiado H, Laizé V, Cancela ML. Matrix Gla protein repression by miR-155 promotes oncogenic signals in breast cancer MCF-7 cells. FEBS Lett. 2016;590: 1234–1241. doi:10.1002/1873-3468.12155

158. Yao J, Guihard PJ, Blazquez-Medela AM, Guo Y, Liu T, Boström KI, et al. Matrix Gla protein regulates differentiation of endothelial cells derived from mouse embryonic stem cells. Angiogenesis. 2016;19: 1–7. doi:10.1007/s10456-015-9484-3

159. Yan T, Lin Z, Jiang J, Lu S, Chen M, Que H, et al. MMP14 regulates cell migration and invasion through epithelial-mesenchymal transition in nasopharyngeal carcinoma. Am J Transl Res. 2015;7: 950–8. Available: http://www.ncbi.nlm.nih.gov/pubmed/26175856

160. Vos MC, Hollemans E, Ezendam N, Feijen H, Boll D, Pijlman B, et al. MMP-14 and CD44 in Epithelial-to-Mesenchymal Transition (EMT) in ovarian cancer. J Ovarian Res. 2016;9: 53. doi:10.1186/s13048-016-0262-7

161. Garmon T, Wittling M, Nie S. MMP14 Regulates Cranial Neural Crest Epithelial-to-Mesenchymal Transition and Migration. Dev Dyn. 2018;247: 1083–1092. doi:10.1002/dvdy.24661

162. Kessenbrock K, Plaks V, Werb Z. Matrix Metalloproteinases: Regulators of the Tumor Microenvironment. Cell. 2010;141: 52–67. doi:10.1016/j.cell.2010.03.015

163. Calvo F, Ege N, Grande-Garcia A, Hooper S, Jenkins RP, Chaudhry SI, et al. Mechanotransduction and YAP-dependent matrix remodelling is required for the generation and maintenance of cancer-associated fibroblasts. Nat Cell Biol. 2013;15: 637–646. doi:10.1038/ncb2756

164. Zhou Y, Li K, Du Y, Wu Z, Wang H, Zhang X, et al. Protein interacting with C-kinase 1 is involved in epithelial-mesenchymal transformation and suppresses progress of gastric cancer. Med Oncol. 2021;38: 34. doi:10.1007/s12032-021-01483-0

165. Liang L, Zeng M, Pan H, Liu H, He Y. Nicotinamide N-methyltransferase promotes epithelial-mesenchymal transition in gastric cancer cells by activating transforming growth factor-β1 expression. Oncol Lett. 2018;15: 4592–4598. doi:10.3892/ol.2018.7885

166. Cui Y, Zhang L, Wang W, Ma S, Liu H, Zang X, et al. Downregulation of nicotinamide N-methyltransferase inhibits migration and epithelial-mesenchymal transition of esophageal squamous cell carcinoma via Wnt/β-catenin pathway. Mol Cell Biochem. 2019;460: 93–103. doi:10.1007/s11010-019-03573-0

167. Hah Y, Cho H, Jo S, Park Y, Heo E, Yoon T. Nicotinamide N‑methyltransferase induces the proliferation and invasion of squamous cell carcinoma cells. Oncol Rep. 2019;42: 1805–1814. doi:10.3892/or.2019.7315

168. Kanska J, Aspuria P-JP, Taylor-Harding B, Spurka L, Funari V, Orsulic S, et al. Glucose deprivation elicits phenotypic plasticity via ZEB1-mediated expression of NNMT. Oncotarget. 2017;8: 26200–26220. doi:10.18632/oncotarget.15429

169. Stagg J, Divisekera U, McLaughlin N, Sharkey J, Pommey S, Denoyer D, et al. Anti-CD73 antibody therapy inhibits breast tumor growth and metastasis. Proc Natl Acad Sci. 2010;107: 1547–1552. doi:10.1073/pnas.0908801107

170. Zhu J, Zeng Y, Li W, Qin H, Lei Z, Shen D, et al. CD73/NT5E is a target of miR-30a-5p and plays an important role in the pathogenesis of non-small cell lung cancer. Mol Cancer. 2017;16: 34. doi:10.1186/s12943-017-0591-1

171. Lupia M, Angiolini F, Bertalot G, Freddi S, Sachsenmeier KF, Chisci E, et al. CD73 Regulates Stemness and Epithelial-Mesenchymal Transition in Ovarian Cancer-Initiating Cells. Stem Cell Reports. 2018;10: 1412–1425. doi:10.1016/j.stemcr.2018.02.009

172. Wang N, Xiang X, Chen K, Liu P, Zhu A. Targeting of NT5E by miR-30b and miR-340 attenuates proliferation, invasion and migration of gallbladder carcinoma. Biochimie. 2018;146: 56–67. doi:10.1016/j.biochi.2017.10.027

173. Sheu C, Chang W, Tsai M, Liao S, Chong I, Kuo P. Bioinformatic analysis of next‑generation sequencing data to identify dysregulated genes in fibroblasts of idiopathic pulmonary fibrosis. Int J Mol Med. 2019;43: 1643–1656. doi:10.3892/ijmm.2019.4086

174. Hesling C, Fattet L, Teyre G, Jury D, Gonzalo P, Lopez J, et al. Antagonistic regulation of EMT by TIF1γ and Smad4 in mammary epithelial cells. EMBO Rep. 2011;12: 665–672. doi:10.1038/embor.2011.78

175. Rowton M, Ramos P, Anderson DM, Rhee JM, Cunliffe HE, Rawls A. Regulation of mesenchymal-to-epithelial transition by PARAXIS during somitogenesis. Dev Dyn. 2013;242: 1332–1344. doi:10.1002/dvdy.24033

176. Steller EJA, Raats DA, Koster J, Rutten B, Govaert KM, Emmink BL, et al. PDGFRB Promotes Liver Metastasis Formation of Mesenchymal-Like Colorectal Tumor Cells. Neoplasia. 2013;15: 204-IN30. doi:10.1593/neo.121726

177. Tang Y-N, Ding W-Q, Guo X-J, Yuan X-W, Wang D-M, Song J-G. Epigenetic regulation of Smad2 and Smad3 by profilin-2 promotes lung cancer growth and metastasis. Nat Commun. 2015;6: 8230. doi:10.1038/ncomms9230

178. Kim M-J, Lee Y-S, Han G-Y, Lee H-N, Ahn C, Kim C-W. Profilin 2 promotes migration, invasion, and stemness of HT29 human colorectal cancer stem cells. Biosci Biotechnol Biochem. 2015;79: 1438–1446. doi:10.1080/09168451.2015.1043118

179. Cui X, Zhang S, Xu Y, Dang H, Liu C, Wang L, et al. PFN2, a novel marker of unfavorable prognosis, is a potential therapeutic target involved in esophageal squamous cell carcinoma. J Transl Med. 2016;14: 137. doi:10.1186/s12967-016-0884-y

180. Yan J, Ma C, Gao Y. MicroRNA-30a-5p suppresses epithelial-mesenchymal transition by targeting profilin-2 in high invasive non-small cell lung cancer cell lines. Oncol Rep. 2017;37: 3146–3154. doi:10.3892/or.2017.5566

181. Zhou K, Chen J, Wu J, Xu Y, Wu Q, Yue J, et al. Profilin 2 Promotes Proliferation and Metastasis of Head and Neck Cancer Cells by Regulating PI3K/AKT/β-Catenin Signaling Pathway. Oncol Res Featur Preclin Clin Cancer Ther. 2019;27: 1079–1088. doi:10.3727/096504019X15579146061957

182. Ling Y, Cao Q, Liu Y, Zhao J, Zhao Y, Li K, et al. Profilin 2 (PFN2) promotes the proliferation, migration, invasion and epithelial-to-mesenchymal transition of triple negative breast cancer cells. Breast Cancer. 2021;28: 368–378. doi:10.1007/s12282-020-01169-x

183. Zhang H, Yang W, Yan J, Zhou K, Wan B, Shi P, et al. Loss of profilin 2 contributes to enhanced epithelial-mesenchymal transition and metastasis of colorectal cancer. Int J Oncol. 2018;53: 1118–1128. doi:10.3892/ijo.2018.4475

184. Lester RD, Jo M, Montel V, Takimoto S, Gonias SL. uPAR induces epithelial–mesenchymal transition in hypoxic breast cancer cells. J Cell Biol. 2007;178: 425–436. doi:10.1083/jcb.200701092

185. Dinh. Downregulation of uPA/uPAR inhibits intermittent hypoxia-induced epithelial-mesenchymal transition (EMT) in DAOY and D283 medulloblastoma cells. Int J Oncol. 2011;38: 733–744. doi:10.3892/ijo.2010.883

186. Laurenzana A, Biagioni A, Bianchini F, Peppicelli S, Chillà A, Margheri F, et al. Inhibition of uPAR-TGFβ crosstalk blocks MSC-dependent EMT in melanoma cells. J Mol Med. 2015;93: 783–794. doi:10.1007/s00109-015-1266-2

187. Semina E V., Rubina KA, Shmakova AA, Rysenkova KD, Klimovich PS, Aleksanrushkina NA, et al. Downregulation of uPAR promotes urokinase translocation into the nucleus and epithelial to mesenchymal transition in neuroblastoma. J Cell Physiol. 2020;235: 6268–6286. doi:10.1002/jcp.29555

188. Gilkes DM, Bajpai S, Chaturvedi P, Wirtz D, Semenza GL. Hypoxia-inducible Factor 1 (HIF-1) Promotes Extracellular Matrix Remodeling under Hypoxic Conditions by Inducing P4HA1, P4HA2, and PLOD2 Expression in Fibroblasts. J Biol Chem. 2013;288: 10819–10829. doi:10.1074/jbc.M112.442939

189. Okumura Y, Noda T, Eguchi H, Sakamoto T, Iwagami Y, Yamada D, et al. Hypoxia-Induced PLOD2 is a Key Regulator in Epithelial-Mesenchymal Transition and Chemoresistance in Biliary Tract Cancer. Ann Surg Oncol. 2018;25: 3728–3737. doi:10.1245/s10434-018-6670-8

190. Wan J, Qin J, Cao Q, Hu P, Zhong C, Tu C. Hypoxia-induced PLOD2 regulates invasion and epithelial-mesenchymal transition in endometrial carcinoma cells. Genes Genomics. 2020;42: 317–324. doi:10.1007/s13258-019-00901-y

191. Larsen JE, Nathan V, Osborne JK, Farrow RK, Deb D, Sullivan JP, et al. ZEB1 drives epithelial-to-mesenchymal transition in lung cancer. J Clin Invest. 2016;126: 3219–3235. doi:10.1172/JCI76725

192. QU H, ZHU M, TAO Y, ZHAO Y. Suppression of peripheral myelin protein 22 (PMP22) expression by miR29 inhibits the progression of lung cancer. Neoplasma. 2015;62: 881–886. doi:10.4149/neo_2015_107

193. Chen Z. The Functional Role of PMP22 Gene in the Proliferation and Invasion of Osteosarcoma. Med Sci Monit. 2015;21: 1976–1982. doi:10.12659/MSM.893430

194. Li X, Jiang H, Xiao L, Wang S, Zheng J. miR-200bc/429 Inhibits Osteosarcoma Cell Proliferation and Invasion by Targeting PMP22. Med Sci Monit. 2017;23: 1001–1008. doi:10.12659/MSM.900084

195. Roux KJ, Amici SA, Fletcher BS, Notterpek L. Modulation of Epithelial Morphology, Monolayer Permeability, and Cell Migration by Growth Arrest Specific 3/Peripheral Myelin Protein 22. Mol Biol Cell. 2005;16: 1142–1151. doi:10.1091/mbc.e04-07-0551

196. Fang F, Flegler AJ, Du P, Lin S, Clevenger C V. Expression of Cyclophilin B is Associated with Malignant Progression and Regulation of Genes Implicated in the Pathogenesis of Breast Cancer. Am J Pathol. 2009;174: 297–308. doi:10.2353/ajpath.2009.080753

197. Zhang X, Tan J, Yang L, An G. Cyclophilin B overexpression predicts a poor prognosis and activates metastatic pathways in colon cancer. Transl Cancer Res. 2020;9: 3573–3585. doi:10.21037/tcr-19-2960

198. Wu H, Xie D, Yang Y, Yang Q, Shi X, Yang R. Ultrasound-Targeted Microbubble Destruction-Mediated miR-206 Overexpression Promotes Apoptosis and Inhibits Metastasis of Hepatocellular Carcinoma Cells Via Targeting PPIB. Technol Cancer Res Treat. 2020;19: 153303382095935. doi:10.1177/1533033820959355

199. Lee Y-S, Jeong S, Kim K-Y, Yoon J-S, Kim S, Yoon K-S, et al. Honokiol inhibits hepatoma carcinoma cell migration through downregulated Cyclophilin B expression. Biochem Biophys Res Commun. 2021;552: 44–51. doi:10.1016/j.bbrc.2021.03.011

200. Sarró E, Durán M, Rico A, Bou-Teen D, Fernández-Majada V, Croatt AJ, et al. Cyclophilins A and B oppositely regulate renal tubular epithelial cell phenotype. Yao X, editor. J Mol Cell Biol. 2020;12: 499–514. doi:10.1093/jmcb/mjaa005

201. Teng M, Huang J, Zhu Z, Li H, Shen J, Chen Q. Cyclophilin B promotes cell proliferation, migration, invasion and angiogenesis via regulating the STAT3 pathway in non-small cell lung cancer. Pathol - Res Pract. 2019;215: 152417. doi:10.1016/j.prp.2019.04.009

202. Ikeda W, Kakunaga S, Takekuni K, Shingai T, Satoh K, Morimoto K, et al. Nectin-like Molecule-5/Tage4 Enhances Cell Migration in an Integrin-dependent, Nectin-3-independent Manner. J Biol Chem. 2004;279: 18015–18025. doi:10.1074/jbc.M312969200

203. Sloan KE, Eustace BK, Stewart JK, Zehetmeier C, Torella C, Simeone M, et al. CD155/PVR plays a key role in cell motility during tumor cell invasion and migration. BMC Cancer. 2004;4: 73. doi:10.1186/1471-2407-4-73

204. Molfetta R, Zitti B, Lecce M, Milito ND, Stabile H, Fionda C, et al. CD155: A Multi-Functional Molecule in Tumor Progression. Int J Mol Sci. 2020;21: 922. doi:10.3390/ijms21030922

205. Morimoto K, Satoh-Yamaguchi K, Hamaguchi A, Inoue Y, Takeuchi M, Okada M, et al. Interaction of cancer cells with platelets mediated by Necl-5/poliovirus receptor enhances cancer cell metastasis to the lungs. Oncogene. 2008;27: 264–273. doi:10.1038/sj.onc.1210645

206. Enloe BM, Jay DG. Inhibition of Necl-5 (CD155/PVR) reduces glioblastoma dispersal and decreases MMP-2 expression and activity. J Neurooncol. 2011;102: 225–235. doi:10.1007/s11060-010-0323-5

207. Zhuo B, Li Y, Gu F, Li Z, Sun Q, Shi Y, et al. Overexpression of CD155 relates to metastasis and invasion in osteosarcoma. Oncol Lett. 2018;15: 7312–7318. doi:10.3892/ol.2018.8228

208. Zheng Q, Gao J, Yin P, Wang W, Wang B, Li Y, et al. CD155 contributes to the mesenchymal phenotype of triple‐negative breast cancer. Cancer Sci. 2020;111: 383–394. doi:10.1111/cas.14276

209. Sato T, Irie K, Ooshio T, Ikeda W, Takai Y. Involvement of heterophilic trans-interaction of Necl-5/Tage4/PVR/CD155 with nectin-3 in formation of nectin- and cadherin-based adherens junctions. Genes to Cells. 2004;9: 791–799. doi:10.1111/j.1365-2443.2004.00763.x

210. Baek JA, Song PH, Ko Y, Gu MJ. High expression of QSOX1 is associated with tumor invasiveness and high grades groups in prostate cancer. Pathol - Res Pract. 2018;214: 964–967. doi:10.1016/j.prp.2018.05.019

211. Zhou L, Chen H-M, Qu S, Li L, Zhao W, Liang Z-G, et al. Reduced QSOX1 enhances radioresistance in nasopharyngeal carcinoma. Oncotarget. 2018;9: 3230–3241. doi:10.18632/oncotarget.23227

212. Katchman BA, Antwi K, Hostetter G, Demeure MJ, Watanabe A, Decker GA, et al. Quiescin Sulfhydryl Oxidase 1 Promotes Invasion of Pancreatic Tumor Cells Mediated by Matrix Metalloproteinases. Mol Cancer Res. 2011;9: 1621–1631. doi:10.1158/1541-7786.MCR-11-0018

213. Geng Y, Xu C, Wang Y, Zhang L. Quiescin Sulfhydryl Oxidase 1 Regulates the Proliferation, Migration and Invasion of Human Glioblastoma Cells via PI3K/Akt Pathway. Onco Targets Ther. 2020;Volume 13: 5721–5729. doi:10.2147/OTT.S255941

214. Toba-Ichihashi Y, Yamaoka T, Ohmori T, Ohba M. Up-regulation of Syndecan-4 contributes to TGF-β1-induced epithelial to mesenchymal transition in lung adenocarcinoma A549 cells. Biochem Biophys Reports. 2016;5: 1–7. doi:10.1016/j.bbrep.2015.11.021

215. Chen L-L, Gao G-X, Shen F-X, Chen X, Gong X-H, Wu W-J. SDC4 Gene Silencing Favors Human Papillary Thyroid Carcinoma Cell Apoptosis and Inhibits Epithelial Mesenchymal Transition via Wnt/β-Catenin Pathway. Mol Cells. 2018;41: 853–867. doi:10.14348/molcells.2018.0103

216. YAN Z, CHEN G, YANG Y, SUN L, JIANG Z, FENG L, et al. Expression and roles of syndecan-4 in dental epithelial cell differentiation. Int J Mol Med. 2014;34: 1301–1308. doi:10.3892/ijmm.2014.1910

217. Fröhling M, Tepasse P, Intemann J, Sambale M, Sherwood J, Paruzel P, et al. Syndecan-4 Modulates Epithelial Gut Barrier Function and Epithelial Regeneration in Experimental Colitis. Inflamm Bowel Dis. 2018;24: 2579–2589. doi:10.1093/ibd/izy248

218. Yuan L, Wu XJ, Li WC, Zhuo C, Xu Z, Tan C, et al. SLC6A8 Knockdown Suppresses the Invasion and Migration of Human Hepatocellular Carcinoma Huh-7 and Hep3B Cells. Technol Cancer Res Treat. 2020;19: 153303382098302. doi:10.1177/1533033820983029

219. Feng Y, Guo X, Tang H. SLC6A8 is involved in the progression of non-small cell lung cancer through the Notch signaling pathway. Ann Transl Med. 2021;9: 264–264. doi:10.21037/atm-20-5984

220. Hall CHT, Lee JS, Murphy EM, Gerich ME, Dran R, Glover LE, et al. Creatine Transporter, Reduced in Colon Tissues From Patients With Inflammatory Bowel Diseases, Regulates Energy Balance in Intestinal Epithelial Cells, Epithelial Integrity, and Barrier Function. Gastroenterology. 2020;159: 984-998.e1. doi:10.1053/j.gastro.2020.05.033

221. Tanno T, Fujiwara A, Sakaguchi K, Tanaka K, Takenaka S, Tsuyama S. Slit3 regulates cell motility through Rac/Cdc42 activation in lipopolysaccharide-stimulated macrophages. FEBS Lett. 2007;581: 1022–1026. doi:10.1016/j.febslet.2007.02.001

222. ZHANG C, GUO H, LI B, SUI C, ZHANG Y, XIA X, et al. Effects of Slit3 silencing on the invasive ability of lung carcinoma A549 cells. Oncol Rep. 2015;34: 952–960. doi:10.3892/or.2015.4031

223. Guan H, Wei G, Wu J, Fang D, Liao Z, Xiao H, et al. Down-Regulation of miR-218-2 and Its Host Gene SLIT3 Cooperate to Promote Invasion and Progression of Thyroid Cancer. J Clin Endocrinol Metab. 2013;98: E1334–E1344. doi:10.1210/jc.2013-1053

224. Bosserhoff A. Slit3 inhibits activator protein 1-mediated migration of malignant melanoma cells. Int J Mol Med. 2011;28: 721–726. doi:10.3892/ijmm.2011.742

225. Zhou W, Gross KM, Kuperwasser C. Molecular regulation of Snai2 in development and disease. J Cell Sci. 2019;132: 1–12. doi:10.1242/jcs.235127

226. Nieto MA. The snail superfamily of zinc-finger transcription factors. Nat Rev Mol Cell Biol. 2002;3: 155–166. doi:10.1038/nrm757

227. Assinder SJ, Stanton J-AL, Prasad PD. Transgelin: An actin-binding protein and tumour suppressor. Int J Biochem Cell Biol. 2009;41: 482–486. doi:10.1016/j.biocel.2008.02.011

228. Dvorakova M, Nenutil R, Bouchal P. Transgelins, cytoskeletal proteins implicated in different aspects of cancer development. Expert Rev Proteomics. 2014;11: 149–165. doi:10.1586/14789450.2014.860358

229. Zhou H, Zhang Y, Chen Q, Lin Y. AKT and JNK Signaling Pathways Increase the Metastatic Potential of Colorectal Cancer Cells by Altering Transgelin Expression. Dig Dis Sci. 2016;61: 1091–1097. doi:10.1007/s10620-015-3985-1

230. Chen Z, He S, Zhan Y, He A, Fang D, Gong Y, et al. TGF-β-induced transgelin promotes bladder cancer metastasis by regulating epithelial-mesenchymal transition and invadopodia formation. EBioMedicine. 2019;47: 208–220. doi:10.1016/j.ebiom.2019.08.012

231. Yu H, Königshoff M, Jayachandran A, Handley D, Seeger W, Kaminski N, et al. Transgelin is a direct target of TGF‐β/Smad3‐dependent epithelial cell migration in lung fibrosis. FASEB J. 2008;22: 1778–1789. doi:10.1096/fj.07-083857

232. Lin Y, Buckhaults PJ, Lee JR, Xiong H, Farrell C, Podolsky RH, et al. Association of the Actin-Binding Protein Transgelin with Lymph Node Metastasis in Human Colorectal Cancer. Neoplasia. 2009;11: 864-IN5. doi:10.1593/neo.09542

233. Lee E-K, Han G-Y, Park HW, Song Y-J, Kim C-W. Transgelin Promotes Migration and Invasion of Cancer Stem Cells. J Proteome Res. 2010;9: 5108–5117. doi:10.1021/pr100378z

234. Chen S. Smad proteins regulate transcriptional induction of the SM22alpha gene by TGF-beta. Nucleic Acids Res. 2003;31: 1302–1310. doi:10.1093/nar/gkg224

235. Qiu P. Interaction of Smad3 and SRF-associated complex mediates TGF-β1 signals to regulate SM22 transcription during myofibroblast differentiation. J Mol Cell Cardiol. 2003;35: 1407–1420. doi:10.1016/j.yjmcc.2003.09.002

236. Nair RR, Solway J, Boyd DD. Expression Cloning Identifies Transgelin (SM22) as a Novel Repressor of 92-kDa Type IV Collagenase (MMP-9) Expression. J Biol Chem. 2006;281: 26424–26436. doi:10.1074/jbc.M602703200

237. Chunhua L, Donglan L, Xiuqiong F, Lihua Z, Qin F, Yawei L, et al. Apigenin up-regulates transgelin and inhibits invasion and migration of colorectal cancer through decreased phosphorylation of AKT. J Nutr Biochem. 2013;24: 1766–1775. doi:10.1016/j.jnutbio.2013.03.006

238. Thompson O, Moghraby JS, Ayscough KR, Winder SJ. Depletion of the actin bundling protein SM22/transgelin increases actin dynamics and enhances the tumourigenic phenotypes of cells. BMC Cell Biol. 2012;13: 1. doi:10.1186/1471-2121-13-1

239. Tsui, Lin, Chang, Hou, Chen, Feng, et al. Transgelin, a p53 and PTEN-Upregulated Gene, Inhibits the Cell Proliferation and Invasion of Human Bladder Carcinoma Cells in Vitro and in Vivo. Int J Mol Sci. 2019;20: 4946. doi:10.3390/ijms20194946

240. Yang B, Chen Q, Wan C, Sun S, Zhu L, Zhao Z, et al. Transgelin Inhibits the Malignant Progression of Esophageal Squamous Cell Carcinomas by Regulating Epithelial–Mesenchymal Transition. Front Oncol. 2021;11: 1–16. doi:10.3389/fonc.2021.709486

241. Polo JM, Anderssen E, Walsh RM, Schwarz BA, Nefzger CM, Lim SM, et al. A molecular roadmap of reprogramming somatic cells into iPS cells. Cell. 2012;151: 1617–1632. doi:10.1016/j.cell.2012.11.039

242. Katoh D, Nagaharu K, Shimojo N, Hanamura N, Yamashita M, Kozuka Y, et al. Binding of αvβ1 and αvβ6 integrins to tenascin-C induces epithelial–mesenchymal transition-like change of breast cancer cells. Oncogenesis. 2013;2: e65–e65. doi:10.1038/oncsis.2013.27

243. Zhang J, Tao T, Wang K, Zhang G, Yan Y, Lin H, et al. IL-33/ST2 axis promotes glioblastoma cell invasion by accumulating tenascin-C. Sci Rep. 2019;9: 20276. doi:10.1038/s41598-019-56696-1

244. Dobie R, West CC, Henderson BEP, Wilson-Kanamori JR, Markose D, Kitto LJ, et al. Deciphering Mesenchymal Drivers of Human Dupuytren’s Disease at Single-Cell Level. J Invest Dermatol. 2022;142: 114-123.e8. doi:10.1016/j.jid.2021.05.030

245. Zhang Y, Zeng W, Xia Y. TWEAK/Fn14 axis is an important player in fibrosis. J Cell Physiol. 2021;236: 3304–3316. doi:10.1002/jcp.30089

246. Perez JG, Tran NL, Rosenblum MG, Schneider CS, Connolly NP, Kim AJ, et al. The TWEAK receptor Fn14 is a potential cell surface portal for targeted delivery of glioblastoma therapeutics. Oncogene. 2016;35: 2145–2155. doi:10.1038/onc.2015.310

247. Wang T, Ma S, Qi X, Tang X, Cui D, Wang Z, et al. Knockdown of the differentially expressed gene TNFRSF12A inhibits hepatocellular carcinoma cell proliferation and migration in vitro. Mol Med Rep. 2017;15: 1172–1178. doi:10.3892/mmr.2017.6154

248. Yang J, Min K-W, Kim D-H, Son BK, Moon KM, Wi YC, et al. High TNFRSF12A level associated with MMP-9 overexpression is linked to poor prognosis in breast cancer: Gene set enrichment analysis and validation in large-scale cohorts. Ahmad A, editor. PLoS One. 2018;13: e0202113. doi:10.1371/journal.pone.0202113

249. Huynh NC-N, Huang T-T, Nguyen CT-K, Lin F-K. Comprehensive Integrated Single-Cell Whole Transcriptome Analysis Revealed the p-EMT Tumor Cells—CAFs Communication in Oral Squamous Cell Carcinoma. Int J Mol Sci. 2022;23: 6470. doi:10.3390/ijms23126470

250. Berzal S, González-Guerrero C, Rayego-Mateos S, Ucero Á, Ocaña-Salceda C, Egido J, et al. TNF-related weak inducer of apoptosis (TWEAK) regulates junctional proteins in tubular epithelial cells via canonical NF-κB pathway and ERK activation. J Cell Physiol. 2015;230: 1580–1593. doi:10.1002/jcp.24905

251. Hu G, Zeng W, Xia Y. TWEAK/Fn14 signaling in tumors. Tumor Biol. 2017;39: 101042831771462. doi:10.1177/1010428317714624

252. Zhao X, Jiang M, Wang Z. TPM4 promotes cell migration by modulating F-actin formation in lung cancer. Onco Targets Ther. 2019;Volume 12: 4055–4063. doi:10.2147/OTT.S198542

253. Jeong S, Lim S, Schevzov G, Gunning PW, Helfman DM. Loss of Tpm4.1 leads to disruption of cell-cell adhesions and invasive behavior in breast epithelial cells via increased Rac1 signaling. Oncotarget. 2017;8: 33544–33559. doi:10.18632/oncotarget.16825

254. Yang R, Zheng G, Ren D, Chen C, Zeng C, Lu W, et al. The clinical significance and biological function of tropomyosin 4 in colon cancer. Biomed Pharmacother. 2018;101: 1–7. doi:10.1016/j.biopha.2018.01.166

255. Chen L, Lin G, Chen K, Liang R, Wan F, Zhang C, et al. VEGF promotes migration and invasion by regulating EMT and MMPs in nasopharyngeal carcinoma. J Cancer. 2020;11: 7291–7301. doi:10.7150/jca.46429

256. Gong J, Zhu S, Zhang Y, Wang J. Interplay of VEGFa and MMP2 regulates invasion of glioblastoma. Tumor Biol. 2014;35: 11879–11885. doi:10.1007/s13277-014-2438-3

257. Vila Ellis L, Cain MP, Hutchison V, Flodby P, Crandall ED, Borok Z, et al. Epithelial Vegfa Specifies a Distinct Endothelial Population in the Mouse Lung. Dev Cell. 2020;52: 617-630.e6. doi:10.1016/j.devcel.2020.01.009

258. Jung EM, Kwon O, Kwon K-S, Cho YS, Rhee SK, Min J-K, et al. Evidences for correlation between the reduced VCAM-1 expression and hyaluronan synthesis during cellular senescence of human mesenchymal stem cells. Biochem Biophys Res Commun. 2011;404: 463–469. doi:10.1016/j.bbrc.2010.12.003

259. Tas F, Karabulut S, Serilmez M, Ciftci R, Duranyildiz D. Clinical significance of serum epithelial cell adhesion molecule (EPCAM) and vascular cell adhesion molecule-1 (VCAM-1) levels in patients with epithelial ovarian cancer. Tumor Biol. 2014;35: 3095–3102. doi:10.1007/s13277-013-1401-z

260. Xia Q, Bai Q-R, Dong M, Sun X, Zhang H, Cui J, et al. Interaction Between Gastric Carcinoma Cells and Neural Cells Promotes Perineural Invasion by a Pathway Involving VCAM1. Dig Dis Sci. 2015;60: 3283–3292. doi:10.1007/s10620-015-3758-x

261. Wang P-C, Weng C-C, Hou Y-S, Jian S-F, Fang K-T, Hou M-F, et al. Activation of VCAM-1 and Its Associated Molecule CD44 Leads to Increased Malignant Potential of Breast Cancer Cells. Int J Mol Sci. 2014;15: 3560–3579. doi:10.3390/ijms15033560

262. Zhang D, Bi J, Liang Q, Wang S, Zhang L, Han F, et al. VCAM1 Promotes Tumor Cell Invasion and Metastasis by Inducing EMT and Transendothelial Migration in Colorectal Cancer. Front Oncol. 2020;10: 1–12. doi:10.3389/fonc.2020.01066

263. Zhou Z, Zhou Q, Wu X, Xu S, Hu X, Tao X, et al. VCAM-1 secreted from cancer-associated fibroblasts enhances the growth and invasion of lung cancer cells through AKT and MAPK signaling. Cancer Lett. 2020;473: 62–73. doi:10.1016/j.canlet.2019.12.039

264. Shu HB, Agranoff AB, Nabel EG, Leung K, Duckett CS, Neish AS, et al. Differential regulation of vascular cell adhesion molecule 1 gene expression by specific NF-kappa B subunits in endothelial and epithelial cells. Mol Cell Biol. 1993;13: 6283–6289. doi:10.1128/mcb.13.10.6283-6289.1993
