# Supplementary material for: Combinatorial targeting of a specific EMT/MET network by macroH2A variants safeguards mesenchymal identity
Source: PLoS One. 2023 Jul 11;18(7):e0288005. doi: 10.1371/journal.pone.0288005 (PMC10335705; doi:10.1371/journal.pone.0288005)
Supplement: S2 Table — Related to S1 Fig. (DOCX) [file pone.0288005.s006.docx]

| shRNA MacroH2A1.1 Probe sequence | 5'-TGACTTCTACACCGGTGGTGAACTCGAGTTCACCACCGGTGTAGAAGTCTTTTTG3' |
| --- | --- |
| shRNA  MacroH2A1.2  Probe Sequence | 5'-GTGAGGTGGAGGCCATAATCAATCTCGAGATTGATTATGGCCTCCACCTCTTTTTG-3' |
| shRNA  MacroH2A2  Probe Sequence | 5'-TGGCCAAACTGGACACCAAGTACTCGAGTACTTGGTGTCCAGTTTGGCCTTTTTG-3’ |
| Scramble  Probe  Sequence | 5’-CCTAAGGTTAAGTCGCCCTCGCTCGAGCGAGGGCGACTTAACCTTAGGTTTTTG-3’ |
